# Mitochondrial complex II orchestrates divergent effects in CD4^+^ and CD8^+^ T cells

**DOI:** 10.1172/JCI194134

**Published:** 2025-12-15

**Authors:** Keisuke Seike, Shih-Chun A. Chu, Yuichi Sumii, Takashi Ikeda, Meng-Chih Wu, Laure Maneix, Dongchang Zhao, Yaping Sun, Marcin Cieslik, Pavan Reddy

**Affiliations:** 1 Dan L. Duncan Comprehensive Cancer Center, Baylor College of Medicine, Houston, Texas, USA.; 2 Department of Hematology and Oncology, Okayama University Hospital, Okayama, Japan.; 3 Department of Pathology and; 4 Department of Computational Medicine & Bioinformatics, University of Michigan School of Medicine, Ann Arbor, Michigan, USA.

**Keywords:** Hematology, Immunology, Metabolism, Bone marrow transplantation, Mitochondria, T cells

## Abstract

Mitochondrial metabolism orchestrates T cell functions, yet the role of specific mitochondrial components in distinct T cell subsets remains poorly understood. Here, we explored the role of mitochondrial complex II (MC II), the only complex from the electron transport chain (ETC) that plays a role in both ETC and metabolism, in regulating T cell functions. Surprisingly, MC II exerts divergent effects on CD4^+^ and CD8^+^ T cell activation and function. Using T cell–specific MC II subunit, succinate dehydrogenase A–deficient (SDHA-deficient) mice, we integrated single-cell RNA-seq and metabolic profiling, with in vitro and in vivo T cell functional assays to illuminate these differences. SDHA deficiency induced metabolic changes and remodeled gene expression exclusively in activated T cells. In CD4^+^ T cells, SDHA loss dampened both oxidative phosphorylation (OXPHOS) and glycolysis, impaired cytokine production, proliferation, and reduced CD4^+^ T cell–mediated graft-versus-host disease after allogeneic stem cell transplantation (SCT). In contrast, SDHA deficiency in CD8^+^ T cells reduced OXPHOS but paradoxically upregulated glycolysis and demonstrated enhanced cytotoxic functions in vitro and in vivo. This metabolic reprogramming endowed SDHA-KO CD8^+^ T cells with superior in vivo antitumor efficacy after immune checkpoint inhibitor therapy and allogeneic SCT. These findings reveal MC II as a bifurcation point for metabolic and functional specialization in CD4^+^ and CD8^+^ T cells.

## Abstract 2

SDHA, a key component of mitochondrial complex II, is a critical bifurcation point in CD4^+^ and CD8^+^ T cell metabolism, function, and differentiation.

## Introduction

T cell activation is a metabolically demanding process that transforms quiescent cells into highly proliferative and functionally specialized effectors (1). Upon stimulation through the T cell receptor (TCR) and costimulatory receptors, quiescent T cells undergo clonal expansion, accompanied by a reprogramming of cellular metabolism to meet the increased bioenergetic and biosynthetic demands of growth and proliferation. While quiescent T cells rely primarily on oxidative phosphorylation (OXPHOS) for ATP production, activated T cells shift to anabolic metabolism, predominantly utilizing glycolysis (2, 3). This metabolic switch is critical for supporting the effector functions of both CD4^+^ and CD8^+^ T cells (1–3). The CD4^+^ and CD8^+^ T cells share functions and pathways, and yet, they also perform distinct tasks upon stimulation by distinct antigenic peptides and TCR stimulation. Although not mutually exclusive, CD4^+^ T cells are the dominant T-helpers, while the CD8^+^ T cells are the cytotoxic effectors. However, the extent to which the metabolic and bioenergetic requirements underpin or shape the dominant functionalities of CD4^+^ and CD8^+^ T cells remains unknown.

Mitochondria are pivotal in regulating T cell activation, proliferation, differentiation, and function (1, 4–6). OXPHOS is indispensable for T cell immunological processes, including antigen-specific T cell activation, the survival and development of memory CD8^+^ T cells, and the suppressive capacity of CD4^+^ Tregs (7–10). The OXPHOS pathway is structured into 5 multiprotein enzyme complexes (complexes I–V) that form the mitochondrial electron transport chain (ETC), utilizing intermediates from the tricarboxylic acid (TCA) cycle to generate ATP (11). The mitochondrial complex II (MC II), or succinate dehydrogenase (SDH), is unique among the ETC complexes because of its dual role in the TCA cycle and ETC (12, 13). Comprising the subunits SDHA, SDHB, SDHC, SDHD, and SDHF, complex II functions as a metabolic and bioenergetic hub (12, 13). Emerging studies have underscored the importance of complex II in CD4^+^ T cell function, yet its precise role in different T cell subsets remains incompletely understood (14, 15). Specifically, it is unclear whether the metabolic and bioenergetic activities of MC II differentially affect the activation and function of CD4^+^ and CD8^+^ T cells.

In this study, we addressed these gaps by examining the role of MC II in CD4^+^ and CD8^+^ T cells using T cell–specific SDHA-KO mice. SDHA deficiency reduced OXPHOS and glycolysis in CD4^+^ T cells, impairing their proliferation and function. Conversely, in CD8^+^ T cells, SDHA deficiency suppressed OXPHOS but unexpectedly upregulated glycolysis that enhanced cytotoxicity. Single-cell RNA-seq (scRNA-seq) revealed that MC II orchestrates distinct metabolic pathways that regulated gene expression programs, cytokine production, and cytotoxicity in these subsets. Functionally, these differential effects attenuated in vivo alloimmune CD4^+^ T cell–mediated graft-versus-host disease (GVHD) while preserving robust in vivo CD8^+^ T cell–mediated antitumor immunity.

Our findings provide insights into the CD4^+^ and CD8^+^ subset-specific roles of MC II in T cell metabolism and function. They redefine our understanding of the role of mitochondria in shaping the unique functionality of the CD4^+^ and CD8^+^ T cells in regulating in vivo adaptive immune responses, such as GVHD and antitumor effects, and offer potential avenues for therapeutic interventions in T cell–mediated immunity.

## Results

### SDHA is a critical regulator of T cell proliferation, cytokine secretion, and oxygen consumption rate.

To delineate baseline differences between unstimulated and stimulated WT T cells, we first took an in vitro reductionist approach. We stimulated splenic CD3^+^ T cells from C57BL/6 (B6) mice using αCD3/αCD28 antibodies in vitro. scRNA-seq was performed on 12,846 cells from unstimulated and stimulated conditions (Supplemental Figure 1A; supplemental material available online with this article; https://doi.org/10.1172/JCI194134DS1), revealing horizontal separation of CD4^+^ and CD8^+^ populations across WT T cells (Supplemental Figure 1B). We performed differential analysis between the stimulated and unstimulated populations (Supplemental Figure 1C) and found multiple metabolic pathways to be affected (Supplemental Figure 1D). As expected, stimulated cells showed increased enrichment of glycolytic processes, generation of diphosphates, and many metabolic processes, indicating a shift in ATP synthesis.

**Figure 1:**
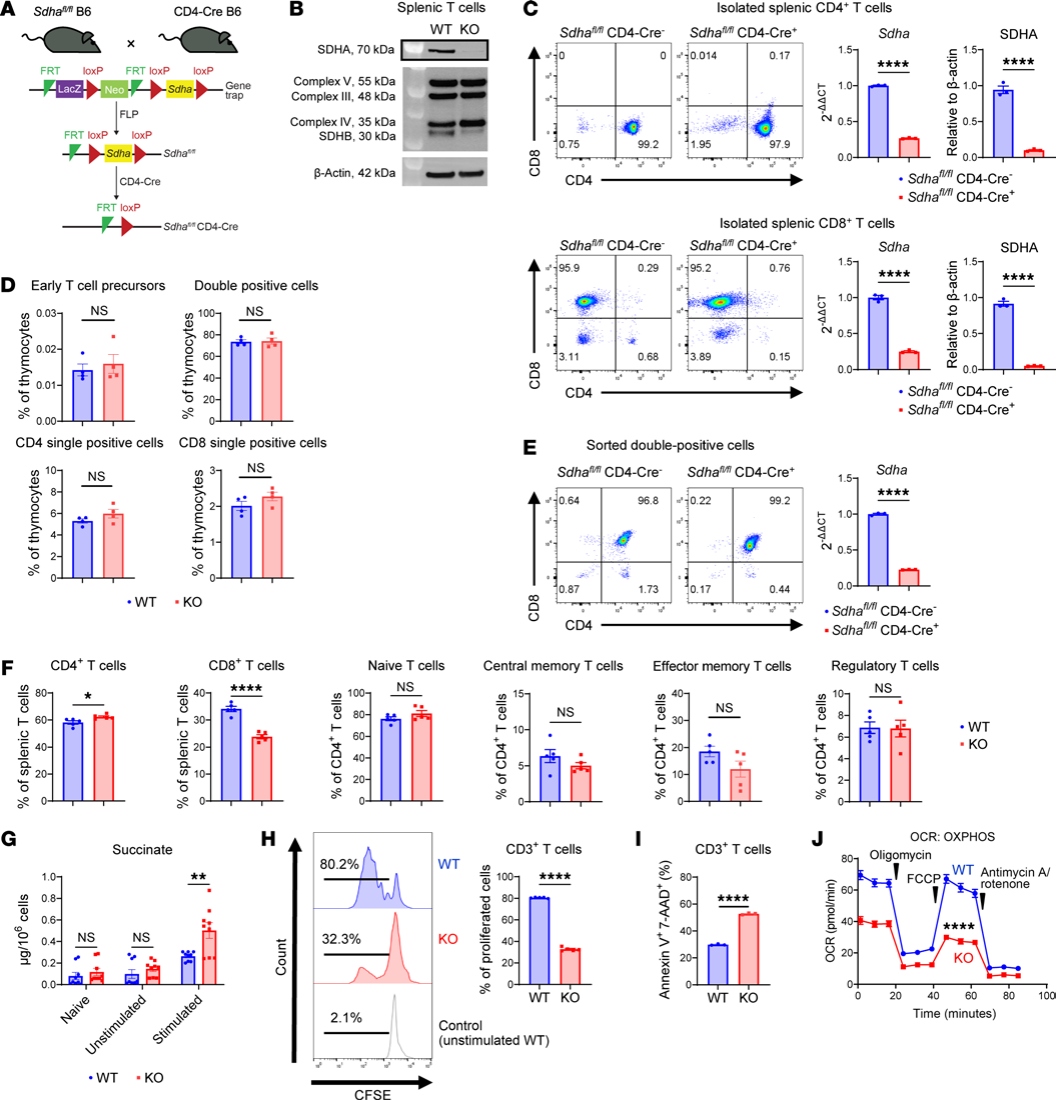
SDHA is required for T cell proliferation and survival following stimulation in vitro. (**A**) *Sdha^fl/fl^* CD4-Cre (SDHA-KO) mice were generated using *Sdha*-floxed mice and CD4-Cre mice. The FRT-flanked region in *Sdha*-floxed mice was excised and crossed with CD4-Cre mice to create *Sdha^fl/fl^* CD4-Cre mice. (**B**) Representative Western blot images of mitochondrial complexes in naive WT and naive SDHA-KO T cells. (**C**) Representative flow cytometry images of isolated splenic CD4^+^ and CD8^+^ T cells, expression of *Sdha* mRNA in CD4^+^ and CD8^+^ T cells from *Sdha^fl/fl^* CD4-Cre^+^ relative to *Sdha^fl/fl^* CD4-Cre^–^ mice, and protein density quantification of SDHA in splenic CD4^+^ and CD8^+^ T cells from *Sdha^fl/fl^* CD4-Cre^–^ and *Sdha^fl/fl^* CD4-Cre^+^ mice (*n* = 3/group). (**D**) The ratio of thymus cell subsets in naive WT and SDHA-KO mouse thymi (*n* = 4/group). (**E**) Representative flow cytometry images of sorted double-positive thymocytes and expression of *Sdha* mRNA in the double-positive cells from *Sdha^fl/fl^* CD4-Cre^+^ relative to *Sdha^fl/fl^* CD4-Cre^–^ mice (*n* = 3/group). (**F**) The ratio of peripheral T cell subsets in naive WT and naive SDHA-KO splenic T cells (*n* = 5/group). (**G**) Succinate levels in WT and SDHA-KO T cells unstimulated and stimulated with CD3/CD28 antibodies for 48 hours or naive WT and SDHA-KO T cells (*n* = 9/group). (**H** and **I**) Proliferative capacity (**H**) and dead cells (**I**) of WT and SDHA-KO T cells, as measured by CFSE staining and 7-AAD^+^ Annexin V^+^ cells after CD3/CD28 antibody stimulation for 48 hours in vitro (*n* = 3-5/group). (**J**) Oxygen consumption rate (OCR, indicator of OXPHOS) in WT and SDHA-KO T cells stimulated by CD3/CD28 antibodies for 2 days, as measured by Seahorse analyzer (*n* = 6–8/group). Two-tailed unpaired *t* test (**C**–**J**) was used to determine significance (mean ± SEM). *P < 0.05, **P < 0.01, ****P < 0.0001.

Given the role of ATP synthesis and OXPHOS in T cell function, we investigated MC II subunit SDHA, a dual regulator of the TCA cycle and ETC. We generated *Sdha^fl/fl^* CD4-Cre (SDHA-KO) mice via Cre-lox recombination and confirmed SDHA deletion through Western blotting (Figure 1, A and B). We further confirmed the deletion of SDHA at the mRNA and protein levels in mature CD4^+^ and CD8^+^ T cells from the spleen (Figure 1C). Notably, SDHA deficiency did not disrupt the expression of other OXPHOS complexes (Figure 1B). We next determined whether deletion of SDHA affected T cell development. We examined thymopoiesis by harvesting thymocytes and analyzing them for early T cell precursors, defined as lineage^–^ (NK-1.1^–^Ly-6G^–^TER-119^–^CD19^–^CD11b^–^TCR γδ^–^) CD4^–^CD8^–^CD25^–^CD44^hi^c-kit^hi^, double-positive (lineage^–^CD4^+^CD8^+^), single-positive (lineage^–^CD4^+^CD8^–^, lineage^–^CD4^–^CD8^+^), and double-negative (lineage^–^CD4^–^CD8^–^) cell subsets (16). The gating strategy is shown in Supplemental Figure 2A. We found that SDHA-KO mice exhibited normal thymopoiesis, with no significant differences in thymic T cell subsets compared with WT mice (Figure 1D and Supplemental Figure 2B). To further confirm the fidelity of the Cre-mediated deletion of the floxed *Sdha* specifically in the thymocytes, we sorted double-positive cells from the thymi and assessed for expression of *Sdha* mRNA. As shown in Figure 1E, double-positive (CD4^+^CD8^+^) cells demonstrated SDHA deficiency. However, splenic T cells in SDHA-KO mice displayed reduced absolute numbers despite maintaining normal subset ratios (Figure 1F and Supplemental Figure 2C).

Next, we compared the functionality of T cells that were deficient for SDHA with the WT T cells. We stimulated them with αCD3/αCD28 and assessed whether deficiency of SDHA led to an increase in its substrate succinate, a metabolite of the TCA cycle. As shown in Figure 1G, SDHA deficiency demonstrated an increase in succinate in SDHA-KO T cells but did not show any change in the MitoTracker Green assay (Supplemental Figure 2D). These data demonstrate that the metabolic consequence of SDHA deficiency is an increase in the intracellular levels of succinate in the KO T cells following their activation. Upon in vitro stimulation with αCD3/αCD28, SDHA-deficient CD3^+^ T cells (excluding dead cells) showed impaired proliferation, as indicated by CFSE dilution (Figure 1H), and increased apoptosis, as indicated by Annexin V and 7-AAD staining when compared with WT T cells (Figure 1I). Furthermore, SDHA-KO CD3^+^ T cells produced elevated levels of intracellular IFN-γ; however, the increase in IFN-γ intracellular level was relatively modest but was also increased at the transcriptomic level in the CD8^+^ T cells (Supplemental Figure 3, A and B). Consistent with SDHA’s role in OXPHOS, SDHA-deficient CD3^+^ T cells exhibited significantly reduced oxygen consumption rates (OCRs) (Figure 1J). These findings demonstrate SDHA as a critical regulator of pan-T cell proliferation, survival, and metabolic reprogramming during activation, but did not affect their development.

### Single-cell analysis demonstrates that SDHA regulates CD4^+^ and CD8^+^ T cell differentiation.

**Figure 2:**
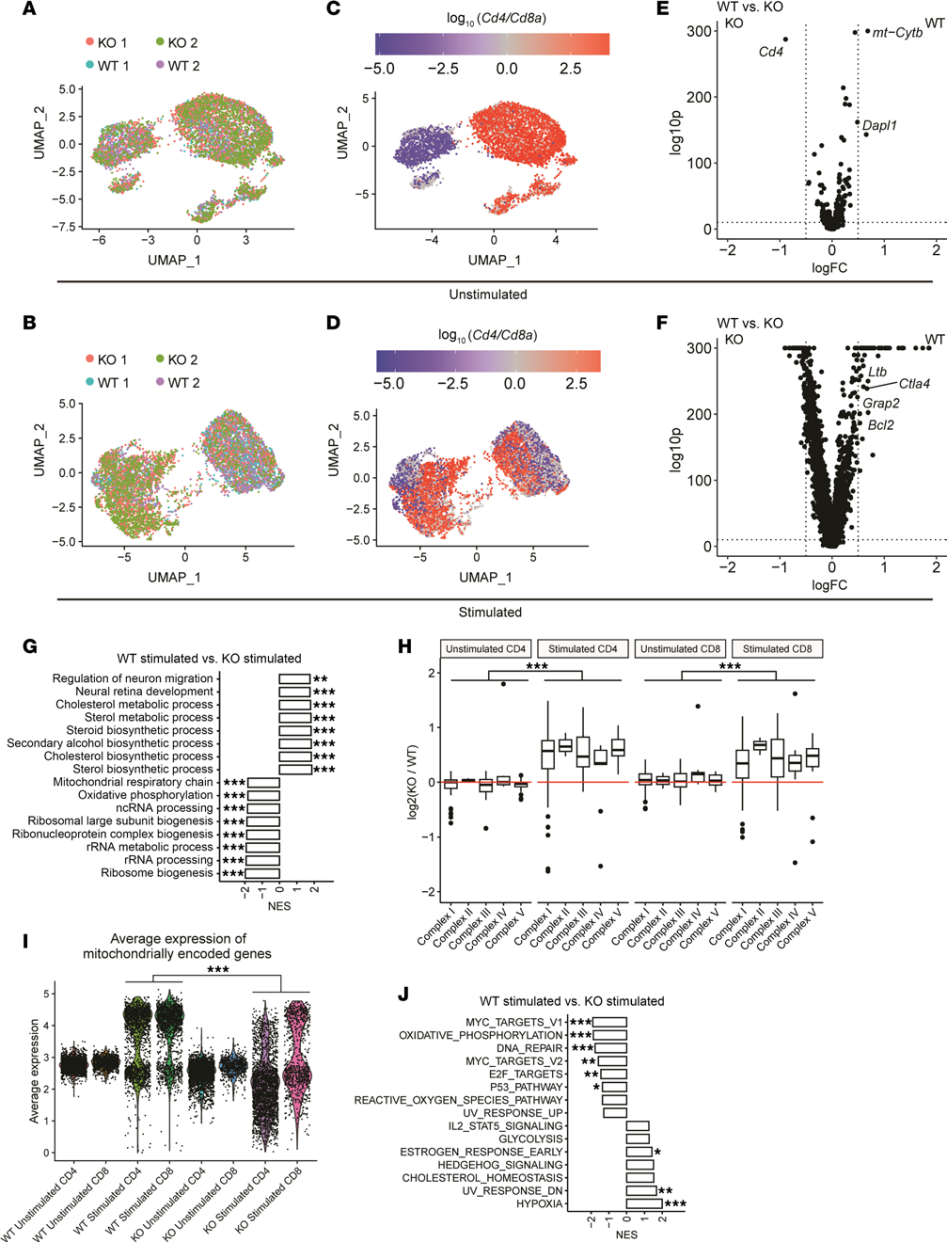
SDHA deficiency resulted in changes of gene signature in T cells after stimulation but not quiescent state. Gene expression of unstimulated or 48-hour-stimulated WT and SDHA-KO T cells, as analyzed by single-cell RNA-seq (scRNA-seq). UMAP embeddings of unstimulated (**A**) and stimulated (**B**) integrated scRNA-seq samples colored by sample source. UMAP embeddings of unstimulated (**C**) and stimulated (**D**) integrated scRNA-seq samples colored by log_2_ ratio of *Cd4*/*Cd8a*. Differential expression between WT and KO T cells in unstimulated (**E**) and stimulated (**F**) conditions. (**G**) Gene Ontology pathway analysis between WT simulated and KO stimulated samples. (**H**) Fold change of mitochondrial complex genes between WT and KO T cells separated by unstimulated vs. stimulated conditions and by CD4 vs. CD8. (**I**) Average expression of mitochondrial genes in CD4^+^ and CD8^+^ T cells across KO, WT, unstimulated, and stimulated conditions (**J**) Hallmark pathway analysis between WT simulated and KO stimulated samples. Permutation tests (**G** and **J**) and Wilcoxon’s test (**H** and **I**) were used to determine significance. *P < 0.05, **P < 0.01, ***P < 0.001.

To gain insights into the role of SDHA, we performed scRNA analysis of cells separated across unstimulated and stimulated conditions (Supplemental Figure 4, A and B). scRNA-seq results were integrated based on the presence of stimulation and the projection of single-cell transcriptomic profiles of T cells, which demonstrated independent clusters reflecting T cell subsets and differentiation states (Figure 2, A and B). Integration of unstimulated cells showed horizontal separation by CD4^+^ and CD8^+^ populations, while stimulated cell integration showed vertical separation of CD4^+^ and CD8^+^ populations (Figure 2, C and D). Differential expression of transcripts between unstimulated SDHA-KO and WT T cells demonstrated a few differentially expressed genes. In contrast, differential expression between stimulated SDHA-KO and WT T cells showed multiple differentially expressed genes (Figure 2, E and F). These data suggested that the metabolic consequence, the subsequent effects on gene expression and functions deficits of SDHA deficiency were more apparent and pronounced following T cell stimulation but not in an unstimulated/quiescent state.

To understand the mechanisms behind changes in gene signature, we performed GSEA pathway analysis to identify pathways activated following T cell stimulation. SDHA-KO T cells showed significant upregulation in pathways related to ribosomal functions and mitochondrial functions while metabolic and biosynthesis processes were downregulated when compared with the WT T cells (Figure 2G). Furthermore, when we compare the expression of mitochondrial subunits (excluding SDHA) in the ETC complexes between unstimulated KO and WT populations, we found that SDHA KO does not affect expression of ETC subunits in unstimulated settings. However, stimulated SDHA-KO cells have much higher expressions of ETC subunits compared with stimulated WT cells in both CD4 and CD8 subsets (Figure 2H and Supplemental Figure 4C). We compared the average expression of the 13 mitochondrially encoded genes. As shown in Figure 2I, the unstimulated WT and SDHA-KO cells showed similar expression of the mitochondrially encoded genes. However, following stimulation, the expression of mitochondrially encoded genes significantly increased in WT CD4^+^ and CD8^+^ T cells (Figure 2I and Supplemental Figure 4D). While the stimulated SDHA-KO CD4^+^ and CD8^+^ T cells showed an increase in expression of these genes when compared with unstimulated SDHA-KO CD4^+^ and CD8^+^ cells, they demonstrated significantly reduced expressions of mitochondrial genes when compared with the stimulated WT T cells. By contrast, the expression of nuclear-coded ETC genes was significantly higher in the stimulated SDHA-KO CD4^+^ and CD8^+^ T cells when compared with the WT CD4^+^ and CD8^+^ T cells (Figure 2H and Supplemental Figure 4E), suggesting a divergence: an increase in the expression of nuclear-coded ETC subunits but a decrease in the mitochondrial genome encoded proteins in the stimulated T cells in the absence of SDHA. Downstream pathway enrichment analysis also revealed different responses between CD4 and CD8 subsets of cells (Figure 2J and Supplemental Figure 4, F and G). The SDHA-KO CD3 as well as the CD4 and CD8 subsets demonstrated enrichment of MYC and OXPHOS pathways. These results indicate that SDHA deficiency leads to significant changes in gene signatures related to mitochondrial metabolic pathways only after T cell stimulation.

**Figure 3:**
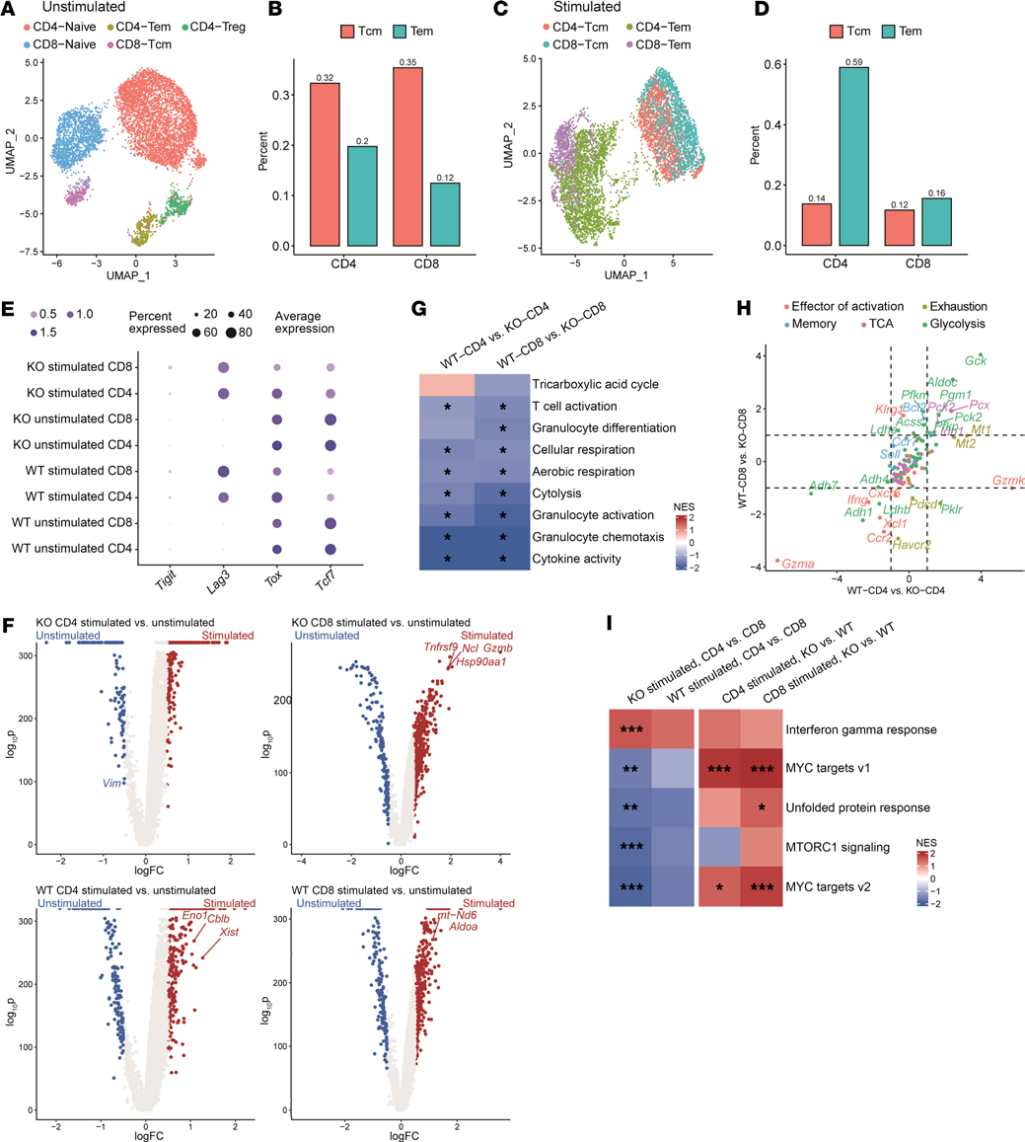
SDHA deficiency induces functional change of CD4^+^ and CD8^+^ T cells through modulating expression of genes associated with T cell activity. Gene expression of unstimulated or 48-hour-stimulated WT and SDHA-KO T cells, as analyzed by scRNA-seq. (**A**) Clustering results and (**B**) percentage of cells identified as Tem or Tcm separated by CD4^+^ or CD8^+^ T cells in unstimulated conditions. (**C**) Clustering results and (**D**) percentage of cells identified as Tem or Tcm separated by CD4^+^ or CD8^+^ T cells in stimulated conditions. (**E**) Expressions of *Tigit, Lag3, Tox*, and *Tcf7* under different conditions. (**F**) Volcano plot showing differential gene expressions across different comparisons. (**G**) Pathway analysis for WT vs. KO in stimulated CD4^+^ and CD8^+^ T cell samples. (**H**) Fold change of gene expression for WT vs. KO in CD4^+^ and CD8^+^ T cells colored by association of genes for pathways or function. (**I**) Pathway analysis for CD4 vs. CD8 in KO and WT stimulated samples as well as KO vs. WT in CD4- and CD8-stimulated samples. Representative pathways were chosen based on pathways that are significant in KO stimulated CD4 vs. CD8, while not significant for WT stimulated CD4 vs. CD8. Permutation test (**G** and **I**) was used to determine significance. *P < 0.05, **P < 0.01, ***P < 0.001.

We next explored whether the changes in gene expression caused by SDHA deficiency led to alterations in T cell differentiation. scRNA-seq was performed on purified splenic T cells from WT and SDHA-KO mice, both unstimulated and stimulated with αCD3/αCD28 antibodies in vitro for 48 hours. We then detected T cell subsets based on gene signatures (Supplemental Figure 5) and categorized them based on expression of *Cd4*, *Cd8a*, naive markers, central memory T cell (Tcm) markers, effector memory T cell (Tem) markers, and Treg markers. In unstimulated conditions naive T cells made up most of the cells, with smaller populations of Tcm, Tem, and Treg in both the WT and SDHA-KO T cells (Figure 3, A and B, and Supplemental Figure 6A). However, after stimulation, we observed much higher proportions of WT CD4^+^ and CD8^+^ Tcm populations (Figure 3, C and D, and Supplemental Figure 6B). We next analyzed whether the cell cycle progression or signatures related to T cell exhaustion (*Tigit*/*Lag3*/*Tox*/*Tcf7*) contributed to the differences in T cell differentiation and found no difference between WT and SDHA KO (Supplemental Figure 6, C and D, and Figure 3E). These results suggest that SDHA deficiency affected both CD4^+^ and CD8^+^ T cell differentiation into subsets of effector and memory T cells after stimulation.

We next hypothesized that the changes in metabolic gene signatures would be distinct between the stimulated WT and SDHA-KO CD4^+^ and CD8^+^ T cells. An overview of differentially expressed genes (Figure 3F) indicated multiple dysregulated genes in different subsets after stimulation. Similar to total T cells, the gene signatures related to aerobic respiration and cellular respiration were downregulated both in WT CD4^+^ and CD8^+^ T cells when compared with SDHA-KO CD4^+^ and CD8^+^ T cells, albeit with CD8^+^ T cells exhibiting significantly greater TCA downregulation (Figure 3G). In addition, SDHA deficiency resulted in changes of several T cell–related pathways, including glycolytic gene expression (Figure 3H) (17). To assess the impact of changes in metabolic pathways to T cell functional changes, we further examined the change in expression of markers related to T cell functions, including activation and exhaustion (Figure 3, E and H). Furthermore, to analyze differences caused by SDHA deficiency between CD4^+^ and CD8^+^ T cells, we identified pathways that were differentially enriched in SDHA KO but not in WT (Figure 3I). Stimulated SDHA-KO CD4^+^ T cells showed upregulation in IFN-γ response; by contrast, SDHA-KO CD8^+^ T cells showed upregulation of MYC and MTORC1 signaling. However, these pathways were significantly enriched when compared with stimulated WT CD4^+^ or CD8^+^ T cells. Thus, SDHA differentially regulates mTOR, MYC, and IFN-γ pathways in T cell subsets.

### SDHA deficiency differentially regulates CD4^+^ and CD8^+^ T cell functions and glycolysis.

**Figure 4:**
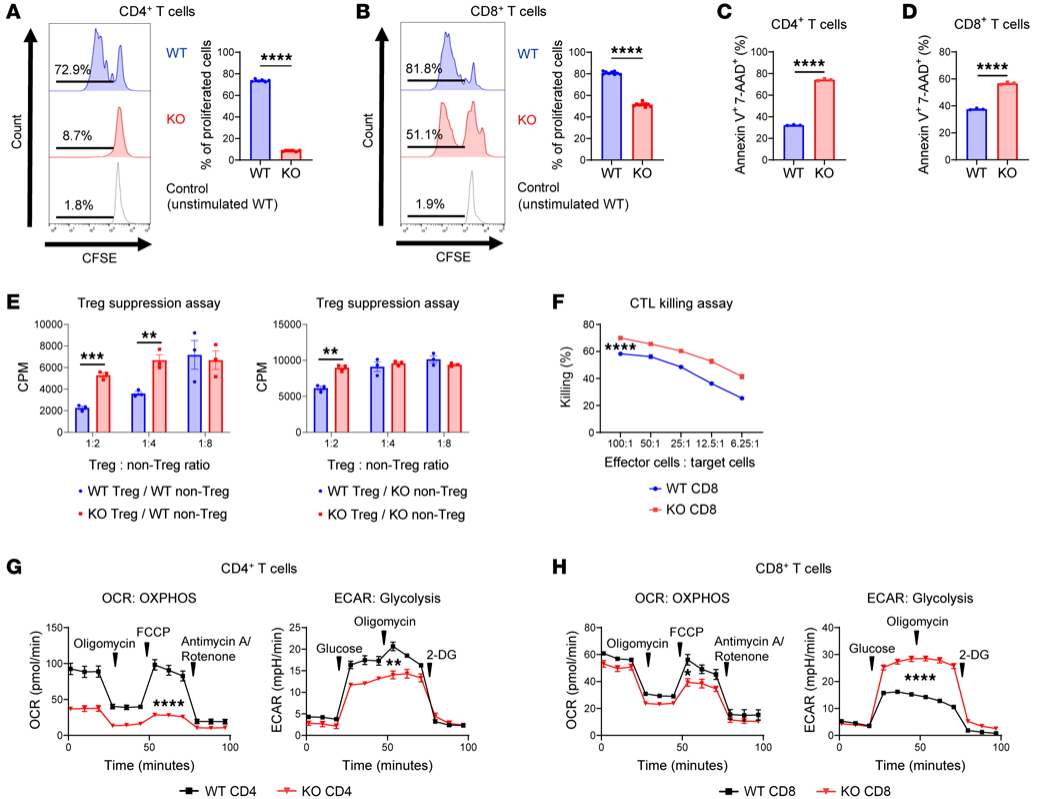
SDHA deficiency leads to different effects on function and bioenergetics between stimulated CD4^+^ and CD8^+^ T cells in vitro. (**A** and **B**) CD4^+^ and CD8^+^ T cells were isolated by bead selection from WT and SDHA-KO mice. CD4^+^ and CD8^+^ T cells stained by CFSE were stimulated by CD3/CD28 antibodies for 48 hours. Proliferation of CD4^+^ (**A**, *n* = 6/group) and CD8^+^ T cells (**B**, *n* = 9/group) as analyzed by flow cytometry. (**C** and **D**) Isolated splenic T cells from either WT or SDHA-KO animals were stimulated by CD3/CD28 antibodies for 48 hours. Annexin V and 7-AAD double-positive CD4^+^ (**C**) and CD8^+^ T (**D**) cells were analyzed by flow cytometry (*n* = 3/group). (**E**) Tregs and non-Tregs were purified by bead selection from WT and SDHA-KO spleen T cells. Tregs were mixed at different ratios with WT or SDHA-KO non-Tregs. Non-Tregs were stimulated by anti-CD3/CD28-coated beads for 72 hours. Proliferation of non-Treg T cells was determined by [^3^H]-thymidine incorporation (*n* = 3/group). (**F**) Isolated splenic T cells from either WT or SDHA-KO animals were cultured with irradiated splenocytes derived from BALB/c for 96 hours, and CD8^+^ T cells were purified as effector cells. A20 cells were used as targets for effector T cells. Results of cytotoxic T lymphocyte (CTL) killing assay with ^51^Cr using effector cells against A20 were analyzed (*n* = 8/group). (**G** and **H**) CD4^+^ (**G**) and CD8^+^ (**H**) T cells from WT and SDHA-KO spleens were stimulated by CD3/CD28 antibodies for 16 hours. OCR and extracellular acidification rate (ECAR, indicator of glycolysis) of CD4^+^ and CD8^+^ T cells were measured by Seahorse analyzer (WT CD4 OCR *n* = 4, KO CD4 OCR *n* = 5, WT CD4 ECAR *n* = 3, SDHA-KO CD4 ECAR *n* = 3, WT CD8 OCR *n* = 3, KO CD8 OCR *n* = 3, WT CD4 ECAR *n* = 4, SDHA-KO CD4 ECAR *n* = 4). Two-tailed unpaired *t* test (**A**–**H**) was used to determine significance (mean ± SEM). **P < 0.01, ***P < 0.001, ****P < 0.0001.

Because glycolysis is critical for T cell functionality and SDHA deficiency impacted the expression of genes in the glycolytic pathway, we next determined whether the SDHA-dependent transcriptional changes in activated T cells impacted their functionality, as determined by Th differentiation and cytotoxicity, as well as proliferation. Both live CD4^+^ and CD8^+^ T cells from SDHA-KO mice demonstrated a reduction in proliferation compared with WT CD4^+^ and CD8^+^ T cells, respectively; but the degree of reduction in proliferation was substantially greater in the SDHA-KO CD4^+^ T cells than in the SDHA-KO CD8^+^ T cells (Figure 4, A and B, and Supplemental Figure 7). However, Annexin V^+^ and 7-AAD^+^ proportions were similarly higher in both stimulated CD4^+^ and CD8^+^ T cells from SDHA-KO mice when compared with those from WT (Figure 4, C and D).

Next, we cultured WT and SDHA-KO CD4^+^ T cells under Th1, Th2, and Th17 polarization conditions. The percentage of IFN-γ–producing and IL-17A–producing CD4^+^ T cells was reduced in SDHA KO under Th1 and Th17 polarization conditions, respectively (Supplemental Figure 8A). By contrast, SDHA-KO CD8^+^ T cells showed similar IFN-γ production (Supplemental Figure 8B). These results demonstrate that SDHA deficiency caused more significant suppression of Th differentiation and proliferation in CD4^+^ T cells but had minimal effect on CD8^+^ T cells proliferation and IFN-γ production.

Because, unlike conventional naive CD4^+^ T cells, Tregs depend on OXPHOS activity (10) for proliferation, we determined the effect of SDHA deficiency on CD4^+^ Tregs to gain further insight. The WT and SDHA-KO Tregs were cocultured with WT or SDHA-KO conventional CD4^+^ non-Tregs (Tcons) and stimulated with αCD3 and αCD28. The SDHA-KO Tregs demonstrated diminished function (Tcon suppression) (Figure 4E). These data demonstrate that SDHA deficiency negatively regulates both CD4^+^ Tcon and CD4^+^ Treg functions.

Next, we analyzed whether cell-intrinsic MC II SDHA deficiency regulated T cell cytotoxic functions. We primed WT and SDHA-KO CD8^+^ T cells with irradiated BALB/c splenocytes and cocultured primed CD8^+^ T cells with BALB/c-derived A20 tumor cells. SDHA-KO CD8^+^ T cells showed higher antitumor cytotoxicity than WT CD8^+^ T cells (Figure 4F), demonstrating that SDHA deficiency promotes greater killing and cytotoxic capacity for CD8^+^ T cells.

To explore the direct link between the functional differences and the metabolic function of SDHA on CD4^+^ and CD8^+^ T cell functions, we next explored the CD4^+^ and CD8^+^ bioenergetic profiles. We analyzed OXPHOS and glycolysis activity in stimulated CD4^+^ and CD8^+^ T cells isolated from the WT and the SDHA-KO splenic T cells to test this. Consistent with the total T cells analysis from above, both SDHA-KO CD4^+^ and CD8^+^ T cells demonstrated reduced OCR (Figure 4, G and H) when compared with the WT CD4^+^ and CD8^+^ T cells, respectively, demonstrating the role of SDHA in both T cell subsets in the regulation of OXPHOS. By contrast, the effect on the extracellular acidification rate (ECAR), a marker of glycolysis, was diametrically opposite. Specifically, while the SDHA-KO CD4^+^ T cells demonstrated reduced ECAR, the SDHA-KO CD8^+^ T cells, by contrast, showed a significant increase in ECAR (Figure 4, G and H).

### Biological and clinical relevance of SDHA deficiency on CD4^+^ and CD8^+^ T cells.

Next, we explored whether SDHA deficiency’s differential effects on the function and glycolysis of CD4^+^ and CD8^+^ T cells were biologically and clinically relevant. Donor T cells drive the toxicity of GVHD and the beneficial graft-versus-tumor (GVT) effect after allogeneic hematopoietic cell transplantation (HCT) (18, 19). Emerging studies have suggested a role for OXPHOS and glycolysis of T cells as promising targets for controlling GVHD (20, 21). Therefore, we first analyzed the effect of SDHA on T cell functional responses to allostimulation in vitro. SDHA-KO T cells stimulated with irradiated splenocytes from allogeneic BALB/c mice showed a significantly reduced proliferation compared with WT T cells, similar to stimulation by αCD3 and αCD28 (Supplemental Figure 9A). We next determined whether this reduction in T cell proliferation was also observed in vivo. To this end, we performed allo-HCT utilizing clinically relevant MHC-mismatched B6 mouse cell into BALB/c bone marrow transplantation (BMT) model, as described in Methods. The in vivo CD4^+^ and CD8^+^ T cell proliferation was tracked by transferring CFSE-stained B6 WT or B6 SDHA-KO T cells into allogeneic BALB/c recipients. Allogeneic SDHA-KO CD4^+^ T cells proliferated significantly less than WT CD4^+^ T cells, whereas allogeneic SDHA-KO CD8^+^ T cells demonstrated similar proliferation compared with WT CD8^+^ T cells (Supplemental Figure 9, B and C).

**Figure 5:**
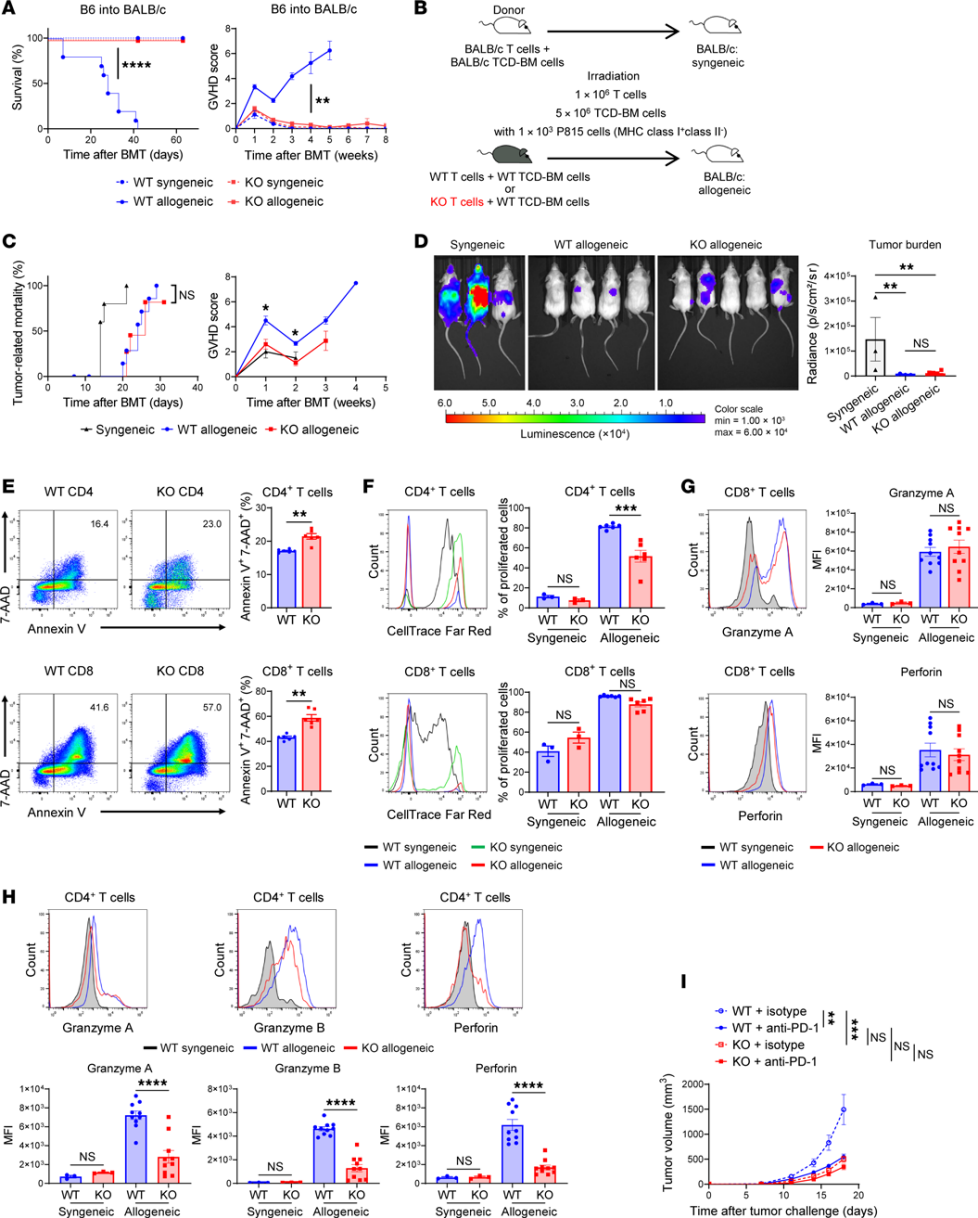
SDHA deficiency in GVHD and GVT effect. (**A**) B6 and BALB/c mice were transplanted with WT or SDHA-KO T cells. Survival rate and clinical GVHD score are shown (syngeneic *n* = 8/group, allogeneic *n* = 10/group). (**B**–**D**) BALB/c mice received HCT with P815 cells (MHC class I^+^class II^–^). (**B**) BALB/c mice received syngeneic or allogeneic T cells from WT or SDHA-KO mice and were infused concurrently with 1.0 × 10^3^ luciferase^+^ P815 cells. (**C**) Tumor-related mortality (syngeneic *n* = 5, allogeneic *n* = 11/group) and clinical GVHD score (*n* = 5/group). (**D**) Representative bioluminescence images and tumor burden on day 14 after HCT (*n* = 3–10/group). (**E**) BALB/c mice received HCT (WT or SDHA-KO B6 → BALB/c). Representative flow cytometry images and Annexin V^+^7-AAD^+^ cells in donor-derived (H-2Kb^+^) CD4^+^ and CD8^+^ T cells day 7 after HCT (*n* = 6/group). (**F**) Representative flow cytometry measuring proliferation of donor-derived (H-2Kb+CD45.2^+^) CD4^+^ and CD8^+^ T cells day 7 after HCT (*n* = 3–6/group). (**G**) Representative flow cytometry images and granzyme A and perforin levels in donor-derived (H-2Kb^+^CD45.2^+^) CD8^+^ T cells day 7 after HCT (*n* = 3–10/group). (**H**) Representative flow cytometry images and granzyme A, granzyme B, and perforin levels in donor-derived (H-2Kb^+^CD45.2^+^) CD4^+^ T cells day 7 after HCT (*n* = 3–10/group). (**I**) WT and SDHA-KO mice were inoculated with B16-F10 melanoma cells on day 0 and injected with anti–PD-1 or isotype control antibody on days 7, 10, 13, and 16. Tumor growth was measured (*n* = 6–9/group). Two-tailed Mann-Whitney *U* test for GVHD score and Annexin V^+^7-AAD^+^, Mantel-Cox log rank test for survival, 1-way ANOVA with Tukey’s post hoc test (**D** and **F**–**H**), and 2-way ANOVA with Tukey’s post hoc test (**I**) were used (mean ± SEM). *P < 0.05, **P < 0.01, ***P < 0.001, ****P < 0.0001.

To examine the clinical relevance of the in vivo effects of SDHA deficiency on CD4^+^ versus CD8^+^ T cells, we determined the effect on GVHD severity utilizing multiple clinically relevant models of allo-HCT. To this end, we specifically utilized well-characterized model systems wherein GVHD is primarily driven by either CD4^+^ T cells or CD8^+^ T cells alone or both subsets contribute to GVHD. We first utilized a well-characterized MHC-mismatched [B6→BALB/c] model wherein CD4^+^ T cells are sufficient to drive GVHD with minor contribution from CD8^+^ T cells (22). Because of the dominant effects of SDHA in regulating CD4^+^ T cell proliferation, differentiation, and cytokine release, we hypothesized that GVHD would be reduced in this model system. Briefly, B6 WT or SDHA-deficient T cells were combined with bone marrow cells from B6 WT mice and transplanted into irradiated allogeneic BALB/c mice, as described in Methods. Consistent with the hypothesis, the recipients of allogeneic SDHA-KO T cells showed a near-complete absence, a striking lack of induction of GVHD, with 0% mortality when compared with allogeneic recipients of B6 WT T cells that showed 100% mortality (Figure 5A).

Although GVHD is closely related to GVT effects after allo-HCT, murine models utilizing MHC class II–deficient but class I^+^ tumors effectively separate CD4^+^ T cell–mediated GVHD from CD8^+^ T cell–mediated GVT (22). Therefore, we next compared the in vivo CD8^+^ T cell–mediated antitumor GVT effects of WT and SDHA-KO B6 CD8^+^ T cells in the context of B6 CD4^+^ T cell–mediated GVHD, by utilizing the above model. The lethally irradiated BALB/c hosts were transplanted with bone marrow cells with either syngeneic BM and T cells from BALB/c donors or from the allogeneic B6 WT mice with either WT or SDHA-KO splenic B6 T cells along with luciferase^+^ P815 cells (express MHC class I but lack MHC class II) that are syngeneic to the BALB/c host. The animals were followed by bioluminescence imaging for tumor burden, as described in Methods (Figure 5B). All of the syngeneic animals demonstrated robust tumor growth and died from tumors, demonstrating a lack of tumor elimination in the absence of alloreactivity. Similar to the above, the allogeneic recipients of SDHA-KO T cells demonstrated little GVHD compared with the allorecipients of B6 WT T cells. However, despite demonstrating similar CD4^+^ mediated GVHD, the allorecipients of SDHA-KO and WT cells still demonstrated similar tumor-related mortality (Figure 5C), demonstrating similar magnitude of preservation of CD8^+^ T cell–mediated GVT effects after allo-HCT (Figure 5D).

We further determined the role of SDHA-deficient donor T cells in reducing GVHD without substantial loss of GVT effects in this HCT model. Allogeneic SDHA-KO CD4^+^ and CD8^+^ T cells demonstrated significantly greater apoptosis in vivo compared with WT T cells (Figure 5E), similar to stimulation in vitro by αCD3 and αCD28. As shown in Figure 5F, no significant differences were observed in proliferation of donor CD4^+^ and CD8^+^ T cells in the syngeneic recipients of WT and SDHA-KO T cells. By contrast, donor CD4^+^ T cells but not CD8^+^ T cells proliferated significantly less in the allogeneic recipients of SDHA-KO T cells than in those of WT T cells (Figure 5F). These data suggest that SDHA deficiency predominantly affected the proliferation of CD4^+^ T cells in vivo and contributed to the reduction in GVHD in addition to causing a bioenergetic collapse that promoted T cell apoptosis.

Because scRNA-seq data showed higher expression of cytotoxicity-relevant genes in the SDHA-KO T cells, we reasoned that preservation of cytotoxicity likely maintained GVT effects in vivo. Therefore, we next analyzed cytotoxic molecules in WT and SDHA-KO T cells after HCT by flow cytometry. Allogeneic SDHA-KO CD8^+^ T cells demonstrated similar levels of granzyme A and perforin when compared with WT CD8^+^ T cells (Figure 5G). However, allogeneic SDHA-KO CD4^+^ T cells showed significantly reduced levels of granzyme A, granzyme B, and perforin when compared with WT CD4^+^ T cells (Figure 5H). These results suggested that SDHA deficiency did not affect its antitumor effects on a per–CD8^+^ T cell basis after allo-HCT, demonstrating the mechanisms for SDHA-KO CD8^+^ T cell–mediated GVT without inducing CD4^+^ T cell–mediated GVHD in vivo.

To rule out possible strain-related artifacts on GVHD and to definitively demonstrate SDHA-dependent negative regulation of CD4^+^ T cells in vivo, we utilized the MHC class II–mismatched B6→B6(C)-*H2-Ab1^bm12^*/KhEgJ (bm12) model (see Methods) wherein GVHD is exclusively driven CD4^+^ T cells (23). The severity of GVHD was significantly reduced in the bm12 recipients of allogeneic SDHA-KO T cells when compared with the bm12 recipients of WT B6 T cells (Supplemental Figure 9D).

Finally, to rule out potential confounding effects of adoptive transfer, and to determine the effect on in vivo antitumor effects of CD8^+^ T cells, we utilized the well-characterized subcutaneous model of B16-F10 melanoma cells that has been extensively utilized to determine the effects of immune checkpoint blockers, as in Methods (24). The B6 WT and the SDHA-KO animals were injected with the tumor cells and with either the anti-PD-1 antibody or rat IgG2a isotype control antibody (see Methods). As expected, the WT B6 animals treated with the anti-PD-1 antibody demonstrated significantly better tumor control than the WT B6 animals treated with the control antibody (Figure 5I). By contrast, the SDHA-KO B6 animals demonstrated significantly better tumor control than the WT B6 control-treated animals, similar to the anti-PD-1-treated WT B6 animals (Figure 5I). Treatment of SDHA-KO B6 animals with anti-PD-1 demonstrated similar tumor control to isotype control antibody-treated KO animals, demonstrating that deficiency of SDHA enhanced CD8^+^ T cell–mediated antitumor immunity to a similar degree as the treatment with anti-PD1 therapy.

## Discussion

Activation of both CD4^+^ and CD8^+^ T cells is associated with pronounced metabolic changes (1, 5). Our data demonstrate that SDHA, a key component of MC II, is a critical bifurcation point in CD4^+^ and CD8^+^ T cell metabolism, function, and differentiation. Through genetic ablation of SDHA in T cells, we revealed that MC II is at the juncture of T cell fate upon activation. While both CD4^+^ and CD8^+^ T cells exhibited reduced OXPHOS following SDHA deletion, their functional responses diverged: the CD4^+^ T cells displayed impaired proliferation and cytokine production and reduced glycolysis along with a reduction in OXPHOS, while the SDHA deficient CD8^+^ T cells paradoxically upregulated glycolysis and enhanced cytotoxic activity while showing a similar reduction on OXPHOS. These findings demonstrate that following activation, mitochondrial bioenergetics do not uniformly govern T cell subsets. Furthermore, our data underscore a function of SDHA in tailoring metabolic circuits to distinct functional demands in the CD4^+^ and CD8^+^ subsets.

Mitochondrial metabolism, specifically OXPHOS, supports memory T cell longevity while glycolysis fuels effector functions (1–3, 8). Our study uncovers a cell-type-specific role for SDHA, a MC II subunit, in shaping these metabolic transitions. In the context of activation, loss of SDHA led to a global suppression of OXPHOS, yet glycolysis was selectively impaired in CD4^+^ T cells while paradoxically enhanced in CD8^+^ T cells. Previous studies have indicated that mitochondrial OXPHOS is essential for CD4^+^ T cell function, particularly in Tregs, where OXPHOS sustains their suppressive capacity (10). In line with this, we observed that SDHA deficiency compromised both conventional CD4 effector and regulatory CD4^+^ T cells, resulting in decreased proliferation, altered memory T cell differentiation, and attenuated cytokine production and Th differentiation. These findings suggest that SDHA is a crucial determinant of the conventional CD4^+^ (Tcon) and Treg immunological fitness and functions.

In addition to performing scRNA analysis, we also expanded our analysis to other methods such as trajectory analysis to better understand how SDHA KO impacts differentiation (Supplemental Figure 10, A–C). We utilized two orthogonal tools, Monocle3 and Slingshot, to calculate cellular trajectory of subtypes across WT and KO cells (Supplemental Figure 10A), as well as across all cells (Supplemental Figure 10, B and C). Although prior cell marker analysis helped us characterize the T cell subsets, trajectory analysis proved challenging to interpret convincingly. Instead of revealing biological progression, the algorithm generated an artifact that separated the T cell states. It is likely that the trajectory was highly dependent on the choice of dimensionality reduction technique and algorithm parameters and is unsuitable when large amounts of variance are tied to mitochondrial gene expression.

Mitochondrial ETC complexes are being increasingly recognized as critical regulators of T cell proliferation and functions (7, 25). We noted strong associations between SDHA-KO and altered mitochondrial gene percentages; however, we found no associations between percentage of mitochondrial gene expressions with proliferation marker *Mki67* (Supplemental Figure 11A) or other proliferation pathways such as E2F and MYC across cell states (Supplemental Figure 11B) (26–28). By contrast, SDHA-deficient CD8^+^ T cells demonstrated a metabolic rewiring that favored glycolysis, leading to increased or retained production of cytotoxic granules, including granzymes and perforin in vitro and in vivo. This metabolic shift correlated with enhanced in vitro cytotoxicity and preservation of CD8^+^-mediated antitumor effects in vivo (GVT and ICI checkpoint therapy) despite reduced OXPHOS activity. These findings align with prior reports demonstrating that glycolysis fuels the cytolytic capacity of effector CD8^+^ T cells (29, 30). A recent study by Chandel and colleagues demonstrated that defective MC III–mediated mitochondrial respiration led to enhanced antiviral immunity and antitumor effects of CD8^+^ T cells (25). Our single-cell transcriptomic analysis revealed that SDHA loss induced a distinct transcriptional profile in CD8^+^ T cells, upregulating genes associated with cytotoxicity while downregulating those linked to OXPHOS. This suggests that SDHA modulates CD8^+^ T cell fate beyond bioenergetics. The potential epigenetic or transcriptional reprogramming mechanisms must be assessed in future studies.

Our findings have significant implications for T cell–mediated immunity in transplantation and cancer therapy. The selective impairment of CD4^+^ T cell proliferation and function in SDHA-deficient mice resulted in a striking reduction in alloreactive CD4^+^-mediated GVHD severity, with recipients of SDHA-KO donor T cells exhibiting near-complete protection from GVHD while retaining potent CD8^+^ T cell–mediated GVT effects. Furthermore, the preservation of cytotoxic molecules in SDHA-KO CD8^+^ T cells, but not in the CD4^+^ T cells, is the likely contributor for the CD8^+^ T cell–mediated GVT effects in vivo. The increase in succinate likely regulates the methylation-dependent transcriptional control of cytotoxic molecules differentially in SDHA-KO CD4^+^ and CD8^+^ T cells. Further studies are needed to address the role of SDHA-mediated methylation programs in regulating cytotoxic molecules in T cells. These observations when taken together with a recent study (15) suggest that the methylation programs regulating DNA accessibility of various cytotoxic molecules may be differentially regulated in different T cell subsets.

Separation of the therapeutic effects of GVT from the side effects of GVHD has been a long-standing challenge in allo-HCT. Remarkably, our data suggest that SDHA inhibition may offer a strategy to selectively suppress pathogenic CD4^+^-dependent GVHD while preserving CD8^+^ T cell–mediated GVT against MHC class I^+^ tumors. The superior CD8^+^ T cell–mediated antitumor effects were also observed in the ICI model of tumor immunity (Figure 5I). Interestingly, the absence of SDHA enhanced CD8^+^ T cell immunity to an extent that is comparable to treatment with anti-PD-1–mediated therapy. This raises the intriguing possibility that SDHA deficiency in CD8^+^ T cells could be a pathway that is either independent or redundant to checkpoint blocker therapy. These alternatives will need to be tested in future studies. Additionally, these observations could have significant implications, beyond alloimmune and antitumor responses, to other T cell–mediated pathologies, such as autoimmunity, allergy, and chronic infections. For example, given that metabolic constraints dictate T cell exhaustion in chronic infections and tumors, it is plausible that SDHA inhibition could reinvigorate exhausted CD8^+^ T cells by promoting glycolytic metabolism and enhancing clearance of chronic infections. Similarly, in autoimmune and allergic diseases that are CD4^+^ T cell–dependent, T-helper polarization, or in MHC class II–restricted diseases, SDHA inhibition could mitigate autoimmune/allergic disorders. Future studies will need to address this plausibility in appropriate model systems.

An interesting aspect of our data is the divergent effects of SDHA deficiency on mitochondria-encoded genome that contributes to ETC and the nuclear genome related to apoptosis, proliferation, and other cellular functions. The genes encoded by mitochondrial genome code for 13 proteins that form only part of the various multiprotein mitochondrial ETC complexes (complex I, III, IV, and V), but not complex II or those related to apoptosis. All of the complex II– and apoptosis-related genes are coded by nuclear genome. Thus, the increase in mitochondrial genome coded expression in the WT is reflective of greater transcription of mitochondrial genome. However, to directly address the concern that a high percentage of mitochondrial transcripts indicates poor cell viability, we have incorporated additional quality control analyses because baseline mitochondrial gene expression is context dependent, varying with tissue type and cellular activation state, and does not necessarily preclude normal cellular function. To demonstrate this within our dataset, we stratified cells into quantiles based on their percentage of mitochondrial gene expression. Our analyses revealed significant positive correlation between high mitochondrial transcript content and the expression of the proliferation marker Mki67. Furthermore, pathway analysis showed depletion for apoptotic gene sets in cells with elevated mitochondrial expression. These findings remain consistent when analyzing CD4^+^ and CD8^+^ T cell subsets independently, supporting the conclusion that the observed mitochondrial gene expression levels are not indicative of apoptosis or compromised cell health in our study. The results suggest that higher mitochondrial gene content in WT cells correlates with elevated MKI67, G2M checkpoint, and inflammatory responses and reduced glycolysis. However, the proliferative differences between mito-gene high and mito-gene low T cells should be carefully determined in future studies.

Our study also raises some questions. Specifically, the dichotomy suggesting SDHA as a metabolic checkpoint might indicate the reliance on distinct fuel sources for CD4^+^ versus CD8^+^ T cell subsets may be context dependent. While our data suggest that SDHA modulates metabolic flux through the TCA cycle and ETC, whether it directly regulates only the qualitative function of mitochondria metabolic enzyme activity or also alters mitochondrial biogenesis and its functions quantitatively warrants deeper investigation. Additionally, given the central role of metabolic intermediates in shaping T cell epigenetics, it will be important to explore whether SDHA deficiency drives the observed functional phenotypes through metabolite mediated chromatin remodeling or transcription factor–orchestrated gene expression programs.

Our study redefines MC II as a critical regulator of CD4^+^ and CD8^+^ T cell metabolism and function following their activation. By uncovering SDHA as a pivotal switch in T cell bioenergetics and differentiation, we provide a conceptual framework for targeting mitochondrial metabolism in immunotherapy and transplantation. These findings open avenues for selectively modulating T cell immunity to enhance antitumor responses while mitigating immunopathology, offering deeper insights into our understanding of mitochondrial control over adaptive immunity.

## Methods

### Sex as a biological variable.

Both sexes were used for all in vitro experiments. For majority of the in vivo BMT experiments female mice were predominantly used as recipients for ease of housing more than 1 mouse per cage. However, the findings are expected to be valid for both sexes.

### Mice.

B6 (H-2K^b^, CD45.2) and BALB/c (H-2K^d^) mice were purchased from Charles River Laboratories. B6.Cg-Tg(Cd4-cre)1Cwi/BfluJ, B6(C)-*H2-Ab1^bm12^*/KhEgJ (bm12), and B6.SJL-*Ptprc^a^ Pepc^b^*/BoyJ (B6 CD45.1, H-2K^b^, CD45.1) mice were purchased from The Jackson Laboratory. The details of *Sdha^fl/fl^* mice were described previously (31). C57BL/6N-*Sdha^tm2a(KOMP)Wtsi^* mice were obtained from Knock Out Mouse Project (KOMP) repository, University of California, and bred to ACTFLPe mice (The Jackson Laboratory) to excise the FRT-flanked region. Then *Sdha^fl/fl^* mice were bred to CD4-Cre mice to create *Sdha^fl/fl^* CD4-Cre (SDHA-KO) mice. Eight- to 12-week-old mice were used for experiments. All mice were kept under specific pathogen–free conditions.

### Cell culture.

Primary murine T cells were purified by magnetic separation (Miltenyi Biotec) from single-cell homogenates of spleens and lymph nodes and cultured in cell media (1640 RPMI supplemented with 10% heat-inactivated FBS, 2 mM L-glutamine, 100 U/mL penicillin-streptomycin, 100 mM HEPES, nonessential amino acids, 1 mM sodium pyruvate, and 50 μM β-mercaptoethanol). T cells were cultured alone or with 5 μg/mL Ultra-LEAF Purified anti-mouse CD3ε (clone 145-2C11, 100359, BioLegend) and 2.5 μg/mL Ultra-LEAF Purified anti-mouse CD28 (clone 37.51, 102121, BioLegend) soluble antibodies for the specified time points. Prior to analysis of cytokine production by intracellular flow cytometry, T cells were additionally stimulated on day 2 with PMA and ionomycin (00-4970-03, Invitrogen) in the presence of Brefeldin A (420601, BioLegend) for 5 hours at 37°C.

For cytokine production of CD4^+^ T cells, isolated CD4^+^ T cells were cultured in the specific cytokine condition. For Th1 polarization, isolated CD4^+^ T cells were cultured for 5 days in an anti-mouse CD3ε (3 μg/mL) precoated plate in the presence of anti-mouse CD28 (3 μg/mL), anti-mouse IL-4 (10 μg/mL, clone 11B11, 504101, BioLegend), recombinant mouse IL-2 (5 ng/mL, 402-ML-020, R&D Systems), and recombinant mouse IL-12 (10 ng/mL, 577002, BioLegend). On day 5, washed CD4^+^ T cells were restimulated in complete medium with 50 ng/mL PMA (P8139, Sigma-Aldrich) and 1 μg/mL ionomycin (I0634, Sigma-Aldrich) in the presence of monensin (420701, BioLegend) for 5 hours.

For Th2 polarization, on day 0, CD4^+^ T cells were cultured in complete medium with Concanavalin A (5 μg/mL, C5275, Sigma-Aldrich), recombinant mouse IL-2 (20 ng/mL), and recombinant mouse IL-4 (50 ng/mL, 574302, BioLegend). On day 3, washed CD4^+^ T cells were cultured in complete medium with recombinant mouse IL-2 (20 ng/mL) and recombinant mouse IL-4 (50 ng/mL). On day 5, CD4^+^ T cells were cultured in an anti-mouse CD3ε (10 μg/mL) precoated plate with complete medium in the presence of anti-mouse CD28 (5 μg/mL) and monensin for 6 hours.

For Th17 polarization, on day 0, CD4^+^ T cells were cultured in an anti-mouse CD3ε (5 μg/mL) precoated plate with complete medium in the presence of anti-mouse CD28 (5 μg/mL), recombinant mouse IL-6 (50 ng/mL, 575704, BioLegend), recombinant human TGF-β1 (1 ng/mL, 781802, BioLegend), recombinant mouse IL-23 (5 ng/mL, 589002, BioLegend), anti-mouse IL-4 (10 μg/mL), and anti-mouse IFN-γ (10 μg/mL, clone XMG1.2, 505833, BioLegend). On day 3, fresh media was slowly added along with the same concentration of antibodies/cytokines as used on day 0. On day 4, washed CD4^+^ T cells were restimulated in complete medium with 500 ng/mL PMA and 500 ng/mL ionomycin in the presence of Brefeldin A for 5 hours.

### Hematopoietic cell transplantation.

Splenic T cells from donors were enriched, and bone marrow cells were depleted of T cells by manual MACS separation (Miltenyi Biotec) utilizing CD90.2 microbeads (130-121-278, Miltenyi Biotec). The details of HCT protocol are described in Supplemental Table 1. The mice were randomly assigned to syngeneic or allogeneic groups in each experiment. No mice were excluded from analysis. No statistical methods were used to predetermine sample size. The investigators were not blinded to allocation during experiments and outcome assessment.

### Systemic analysis of GVHD.

Survival after HCT was monitored daily and assessed the degree of clinical GVHD weekly, as described previously (32).

### Induction of tumor and bioluminescence imaging.

P815 leukemia cells (catalog TIB-64) were purchased from ATCC and were transduced with luciferase (33). On day 0, 1.0 × 10^3^ luciferase^+^ P815 cells (H-2K^d^) were injected into each recipient along with syngeneic (BALB/c) or allogeneic (B6) TCD-BM and T cells. Bioluminescence imaging was performed with a cryogenically cooled CCD camera (IVIS, Caliper Life Sciences). Mice were intraperitoneally injected with 150 μg/body weight (g) D-Luciferin (LUCK-100, GoldBio) 10 minutes before imaging. Survival was monitored daily, and we attributed death to tumor if tumor was present at the necropsy and/or hind-leg paralysis.

### B16-F10 melanoma model.

Mice were injected subcutaneously with 2.0 × 10^5^ B16-F10 melanoma cells (CRL-6475, ATCC) in the right flank (24). Tumors were measured regularly with calipers, and tumor volumes were calculated using the following formula: length × width × [(length × width)^0.5^] × π/6. Anti-PD-1 antibody (200 μg, clone RMP1-4, BE0146, Bio X Cell) or rat IgG2a isotype control antibody (200 μg, clone 2A3, BE0089, Bio X Cell) was injected intraperitoneally in 100 μL PBS on days 7, 10, 13, and 16 after tumor injection. Mice were euthanized when tumors reached a size of 15 mm in diameter or ulcer formation occurred.

### Cell isolation and quantitative RT-PCR.

Murine CD4^+^CD8^+^ double-positive cells were isolated from single-cell homogenates of thymi using the FACSAria II (BD Biosciences) and the Sony MA900 (Sony Biotechnology). Mature peripheral CD4^+^ and CD8^+^ T cells were isolated from single-cell homogenates of spleens using the CD4^+^ T Cell Isolation Kit (130-104-454, Miltenyi Biotec), CD8 MicroBeads (130-117-044, Miltenyi Biotec), and manual MACS separation (Miltenyi Biotec). Total RNA was isolated from these single-cell suspensions with the RNeasy Kit (74136, QIAGEN). The iTaq Universal SYBR Green One-Step Kit (1725151, Bio-Rad) and the following primers were used to detect the following transcripts: 5′-ACATGCAGAAGTCGATGCAG-3′ and 5′-CATTCCCCTGTCGAATGTCT-3′ (*Sdha*); 5′-GAGCTGTTTGCAGACAAAGTTC-3′ and 5′-CCCTGGCACATGAATCCTGG-3′ (*Ppia*). All reactions were performed according to the manufacturer’s instructions. All primers were verified for the production of a single specific PCR product via melting curve analysis.

### Immunoblot analysis.

T cells were lysed in RIPA buffer (89901, Thermo Fisher Scientific or R0278, Sigma-Aldrich). Equal amounts of proteins were loaded on 4%–12% SDS-PAGE gel (NP0321, Thermo Fisher Scientific) or Mini-PROTEAN TGX Stain-Free Precast Gels (4568124, Bio-Rad), electrophoresed and subsequently transferred to a PVDF membrane (ISEQ85R, Sigma-Aldrich or 1704156, Bio-Rad) using a Bio-Rad semi-dry transfer cell (20 V, 1 h) or a Trans-Blot Turbo Transfer System (120 V, 1 h). Blots were incubated with mouse anti-SDHA (clone 2E3GC12FB2AE2, ab14715, Abcam), rabbit anti-SDHA (clone EPR9043(B), ab137040, Abcam), total OXPHOS rodent antibody cocktail (ab110413, Abcam), mouse anti-β actin (clone mAbcam 8226, ab8226, Abcam), and rabbit anti-β actin (clone HL1926, GTX637675, GeneTex) primary antibodies overnight at 4°C. Incubation with secondary anti-rabbit-HRP (SA002, GenDEPOT) and anti-mouse-HRP (sc-516102, Santa Cruz Biotechnology) was performed at room temperature for 1 hour. Bound antibody was detected using SuperSignal ECL substrate (Thermo Fisher Scientific) or Clarity Western ECL Substrate (Bio-Rad) and quantitated using ChemiDoc MP Imaging system (Bio-Rad).

### Succinate quantification.

Isolated T cells were collected, processed, and analyzed using the Succinate Assay Kit (ab204718, Abcam) according to the manufacturer’s protocol.

### Seahorse analysis.

Analysis was performed on T cells isolated from WT and SDHA-KO mice. Cells were washed 3 times in complete seahorse XF assay medium (103335-100, Agilent Technologies) with 17.5 mM glucose, 1 mM sodium pyruvate, 2 mM glutamine, 2% BSA 10 μM Y-27632, and 1% penicillin-streptomycin adjusted to pH 7.4. Cells were plated at 1 × 10^5^ cells per well in a Seahorse assay plate, pretreated with Cell-Tak (354240, Corning). Cells were equilibrated to 37°C for 30 minutes before assay. Respiration profile (OCR) was assessed in 96XF instrument with Mitostress assay as indicated upon cell treatment with oligomycin (75351, 2 μM, Sigma-Aldrich), carbonyl cyanide 4-(trifluoromethoxy)phenylhydrazone (FCCP, C2990, 10 μM, Sigma-Aldrich), rotenone (R8875, 1 μM, Sigma-Aldrich), and antimycin A (A8674, 10 μM, Sigma-Aldrich) (34). Glycolytic function (ECAR) was assessed in 96XF instrument with Glycolysis stress test as indicated upon cell treatment with glucose (10 mM, G8270, Sigma-Aldrich), oligomycin (75351, 2 μM, Sigma-Aldrich), FCCP (C2990, 10 μM, Sigma-Aldrich), rotenone (R8875, 1 μM, Sigma-Aldrich), and 2-Deoxy-D-glucose (2-DG, D8375, 50 mM, Sigma-Aldrich).

### Cytotoxic T lymphocyte killing assay.

Splenic T cells from WT B6 and SDHA-KO animals and irradiated (30 Gy) red blood cell lysed splenocytes from WT BALB/c mice were cocultured in 5:2 ratio for 96 hours. Splenic CD8^+^ T cells were isolated from above cocultured cells, purified with CD8 MicroBeads (130-117-044, Miltenyi Biotec), and used as effector cells. A20 cells (TIB-208, ATCC) were incubated with 2 MBq of Na_2_^51^CrO_4_ (NEZ030001MC, PerkinElmer) for 2 hours at 37°C in a 5% CO_2_ atmosphere and were used as target cells. After washing, 4 × 10^3^ labeled targets were resuspended and added to triplicate wells at varying effector-to-target ratios and then incubated for 4 hours. Maximal and minimum release was determined by the addition of Triton-X or media alone to targets, respectively. After incubation, supernatants were transferred to a LumaPlate (600633, PerkinElmer), and ^51^Cr activity was determined using TopCount NXT (Hewlett Packard).

### Treg suppression assay and [^3^H]-thymidine incorporation.

The Treg suppression assay protocol was described previously (35). Purified Tregs were isolated from WT and SDHA-KO T cells by using CD4^+^CD25^+^ Regulatory T Cell Isolation Kit (130-091-041, Miltenyi Biotec). Non-Tregs were isolated by depleting Tregs from WT and SDHA-KO T cells. Treg and non-Tregs (50,000 cells/well) were added to triplicate wells at varying Treg/non-Treg ratios. The same number of Dynabeads Mouse T-Activator CD3/CD28 (11452D, Thermo Fisher Scientific) as T cells were to all wells. Plates were incubated at 37°C, 5% CO_2_ for 72 hours. Plates were pulsed with 0.1 μCi [^3^H]-thymidine (NET027005MC, PerkinElmer) per well 8 hours prior to completion of experiment. The incorporation of [^3^H]-thymidine by proliferating T cells during the final 8 hours of culture was measured using TopCount NXT (Hewlett Packard).

### Flow cytometry.

Flow cytometric analysis was performed using the following antibodies and reagents: APC anti-mouse CD3 (clone 17A2, 100236, 1:200, BioLegend), PerCP/Cyanine5.5 anti-mouse CD3 (clone 17A2, 65-0032-U100, 1:100, Tonbo Biosciences), PerCP/Cyanine5.5 anti-mouse CD4 (clone GK1.5, 100434, 1:200, BioLegend), PE/Cyanine7 anti-mouse CD4 (clone GK1.5, 100422, 1:200, BioLegend), violetFluor 450 anti-mouse CD4 (clone GK1.5, 75-0041-U100, 1:100, Tonbo Biosciences), APC anti-mouse CD8a (clone 53-6.7, 100712, 1:200, BioLegend), APC/Cy7 anti-mouse CD8a (clone 53-6.7, 100714, 1:200, BioLegend), PerCP/Cyanine5.5 anti-mouse CD8a (clone 53-6.7, 100734, 1:100, BioLegend), PE anti-mouse TCR β chain (clone H57-597, 109208, 1:200, BioLegend), FITC anti-mouse CD25 (clone 3C7, 101908, 1:200, BioLegend), APC anti-mouse CD117 (c-kit) (clone ACK2, 135108, 1:200, BioLegend), PE/Cyanine7 anti-mouse/human CD44 (clone IM7, 103030, 1:200, BioLegend), FITC anti-mouse/human CD44 (clone IM7, 103022, 1:200, BioLegend), PE anti-mouse CD62L (clone W18021D, 161204, 1:200, BioLegend), PE anti-mouse FOXP3 (clone MF-14, 126404, 1:100 BioLegend), PE/Cyanine7 anti-mouse H-2K^b^ (clone AF6-88.5, 116520, 1:100, BioLegend), violetFluor 500 anti-mouse CD45.2 (clone 104, 85-0454-U100, 1:100, Tonbo Biosciences), PE anti-mouse Granzyme A (clone 3G8.5, 149704, 1:200, BioLegend), FITC anti-human/mouse Granzyme B (clone GB11, 515403, 1:100, BioLegend), PE anti-mouse Perforin (clone S16009A, 154404, 1:100, BioLegend), PE anti-mouse IL-4 (clone 11B11, 504104, 1:100, BioLegend), APC anti-mouse IFN-γ (clone W18272D, 163514, 1:100, BioLegend), PE anti-mouse IFN-γ (clone XMG1.2, 505808, 1:100, BioLegend), FITC anti-mouse IL-17A (clone TC11-18H10.1, 506908, 1:100, BioLegend), PE anti-mouse NK-1.1 (clone PK136, 108708, 1:200, BioLegend), FITC anti-mouse Ly-6G (clone 1A8, 127606, 1:200, BioLegend), PE anti-mouse Ly-6G (clone 1A8, 127608, 1:200, BioLegend), PE anti-mouse TER-119 (clone TER-119, 116208, 1:200, BioLegend), PE anti-mouse CD19 (clone 1D3/CD19, 152408, 1:200, BioLegend), PE anti-mouse/human CD11b (clone M1/70, 101208, 1:200, BioLegend), FITC anti-mouse TCR γ/δ (clone GL3, 118105, 1:200, BioLegend), PE anti-mouse TCR γ/δ (clone UC7-13D5, 107508, 1:200, BioLegend), 7-AAD (420404, 1:100, BioLegend), PE Annexin V (640947, 1:500, 1:20, Biolegend), Zombie NIR (423106, 1:5,000, BioLegend), CFSE (C34570, Thermo Fisher Scientific), CellTrace Far Red (C34572, Thermo Fisher Scientific), MitoTracker Green (M46750, Thermo Fisher Scientific), Cyto-Fast Fix/Perm Buffer Set (426803, BioLegend), and Foxp3/Transcription Factor Staining Buffer Set (00-5523-00, Invitrogen) according to the manufacturer’s protocol. For proliferation studies, only live cells (7-AAD or Zombie NIR negative) were assessed. Cells were run on the Attune NxT flow cytometer or the Cytek Northern Lights and analyzed using FlowJo v10.2.

### Single-cell RNA sequence of T cells.

WT and SDHA-KO splenic T cells were simulated by anti-CD3/CD28 antibodies for 48 hours. Unstimulated and stimulated T cells were utilized for scRNA-seq after removing dead cells by Dead Cell Removal Kit (130-090-101, Miltenyi Biotec). Single-cell suspensions were subjected to final cell counting on the LUNA-FX7 Automated Cell Counter (Logos Biosystems) and diluted to a concentration of 700–1,000 cells/μL. 3′ single-cell libraries were generated using the 10x Genomics Chromium Controller and following the manufacturer’s protocol for 3′ HT v3.1 chemistry (10x Genomics). Final library quality was assessed using the LabChip GXII HT (PerkinElmer), and libraries were quantified by Qubit (Thermo Fisher Scientific). Pooled libraries were subjected to 28 × 10 × 10 × 151 bp paired-end sequencing according to the manufacturer’s protocol (NovaSeq 6000, Illumina). Bcl2fastq2 Conversion Software (Illumina) was used to generate demultiplexed Fastq files, and the Cell Ranger Pipeline (10x Genomics) was used to align reads and generate count matrices. Single-cell analysis was performed using the Seurat package (4.2.0) using the standard protocol (36). Cells with more than 6,000 features for unstimulated or more than 8,000 features in stimulated samples were filtered. Data normalization was performed using log1p normalization, and integration was performed to merge data. Clustering was performed using Seurat’s unsupervised graph-based clustering approach. An initial clustering was performed to identify clusters and filter those without *Cd3g, Cd3d, or Cd3e* expression. This was followed by a second reclustering to identify and categorize cluster phenotype. Assignment of CD4^+^ and CD8^+^ clusters were performed by taking the log ratios of *Cd4*/*Cd8a*. Tem, Tcm, naive, and Treg clusters were identified by comparing expressions of *Cd44*, *Sell*, *Itga4*, *Ccr7*, *Il7r*, *Tcf7*, and *Foxp3*.

### Pathway analysis.

For functional characterization of clusters, pathway analysis was performed using gene set enrichment analysis (GSEA) (37). We calculated normalized enrichment scores (NES) from fold changes between clusters for C5 biological pathway gene sets to identify affected cellular components. We used the GSEA implementation via clusterprofiler (version 4.9.0) (38). Additional analysis of genes involved in the ETC complexes and subunits were derived from the Gene Ontology knowledgebase.

### Trajectory analysis.

scRNA-seq trajectory analysis was performed using both Monocle3 (https://cole-trapnell-lab.github.io/monocle3/) and Slingshot (https://github.com/kstreet13/slingshot) to infer cellular differentiation paths. Monocle3 was applied to WT subsets, KO subsets, or total cells using default parameters. Trajectories and cell states were visualized using Monocle3’s plotting functions, with colors indicating the cell subtypes. Slingshot was also applied using default parameters and a resolution of 1.5 for clustering. Trajectories were learned with the option for multiple or branching paths, and final results were visualized using slingshot’s plotting functions.

### Statistics.

Experiments were conducted with technical and biological replicates at an appropriate sample size, as estimated by our prior experience. No statistical methods were used to predetermine sample size. No methods of randomization and no blinding were applied. All data were replicated independently at least once as indicated in the figure legends, and all attempts to reproduce experimental data were successful. Bars and error bars represent the mean ± SEM, respectively. All statistical analysis was performed using Graph Pad Prism 9. *P* values of less than 0.05 were considered as significant; *P* values of more than 0.05 were considered as not significant. All sample sizes and statistical tests used are detailed in each figure legend.

### Study approval.

All animal work was performed according to regulations reviewed and approved by the Institutional Animal Care and Use Committee of the University of Michigan (PRO00009494) or Baylor College of Medicine (AN-8909).

### Data availability.

Raw and processed sequencing data generated as part of this study are available through the Gene Expression Omnibus with accession number GSE307567. The values of all data points in graphs are reported in the Supporting Data Values file.

## Author contributions

KS, SCAC, and Y Sumii designed and performed experiments, analyzed the data, and wrote the manuscript. TI, MCW, LM, DZ, and Y Sun performed experiments. PR conceived and designed the study. MC and PR planned and guided the research and wrote the manuscript. MC and PR supervised the study.

## Funding support

This work is the result of NIH funding, whole or in part, and is subject to the NIH Public Access Policy. Through acceptance of this federal funding, the NIH has been given a right to make the work publicly available in PubMed Central.

- NIH grants P01CA039542, P01HL149633, R01HL152605, and R01AI165563.
- Cancer Prevention and Research Institute of Texas RR220033 to PR.

## Supplementary Material

Supplemental data

Unedited blot and gel images

Supporting data values

## Version 1 12/15/2025

Electronic publication
