# Supplementary material for: Mitochondrial complex II orchestrates divergent effects in CD4+ and CD8+ T cells
Source: J Clin Invest. 2025 Dec 15;135(24):e194134. doi: 10.1172/JCI194134 (PMC12700539; doi:10.1172/JCI194134)
Supplement: Supplemental data [file jci-135-194134-s083.pdf]

Supplemental Figure 1

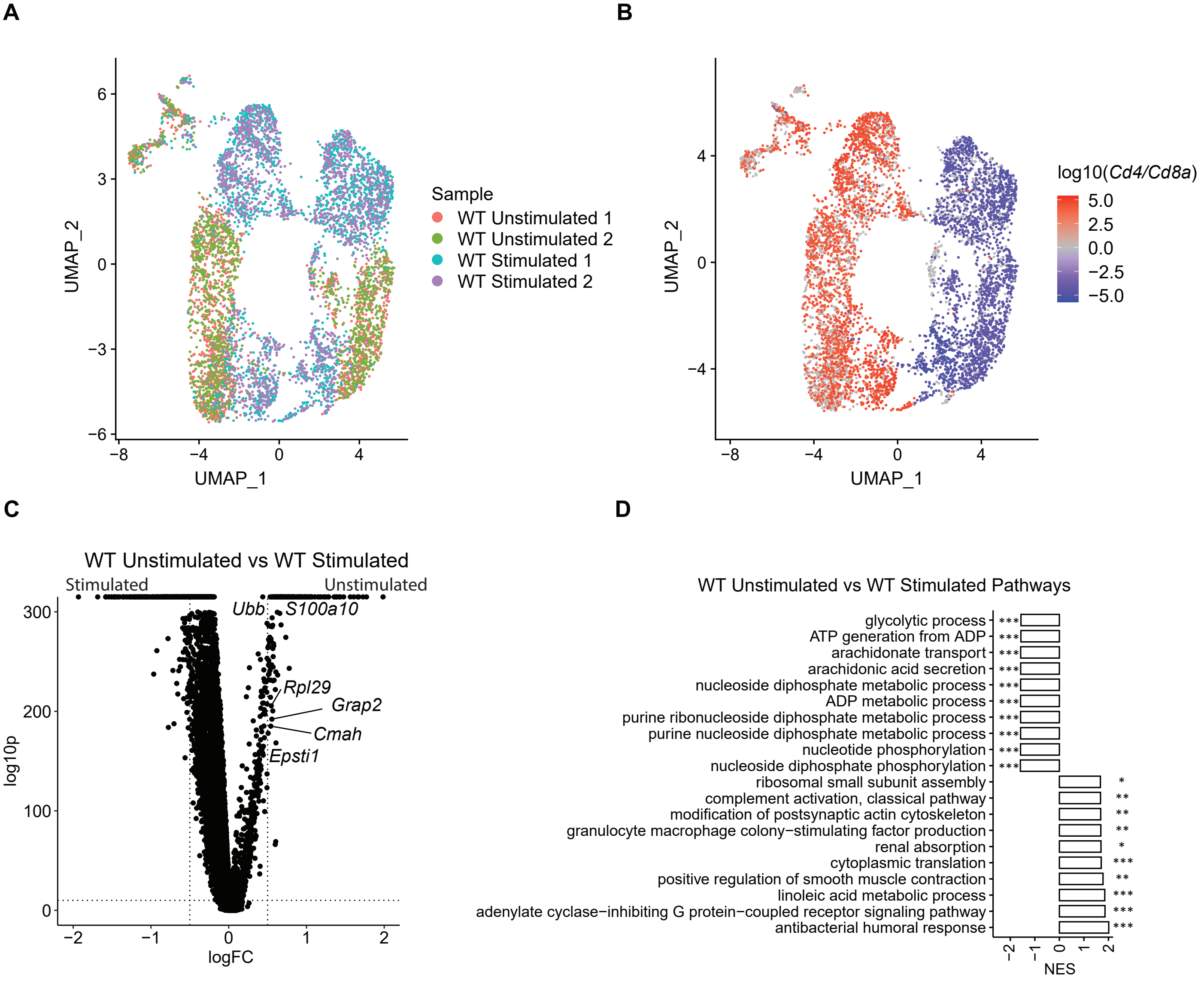

Supplemental Figure 1. WT stimulated and unstimulated scRNA-seq.

Gene expression of unstimulated or 48 h stimulated WT T cells were analyzed by single cell RNA-seq (scRNA-seq). (**A**) UMAP embeddings of integrated scRNA-seq samples colored by sample source. (**B**) UMAP embeddings of integrated scRNA-seq samples colored by log2 ratio of *Cd4*/*Cd8a*. (**C**) Differential expression between stimulated and unstimulated condition. (**D**) Pathway analysis between stimulated and unstimulated samples. Permutation test (**D**) was used to determine significance. $^*P < 0.05$, $^{**}P < 0.01$, $^{***}P < 0.001$.

Supplemental Figure 2

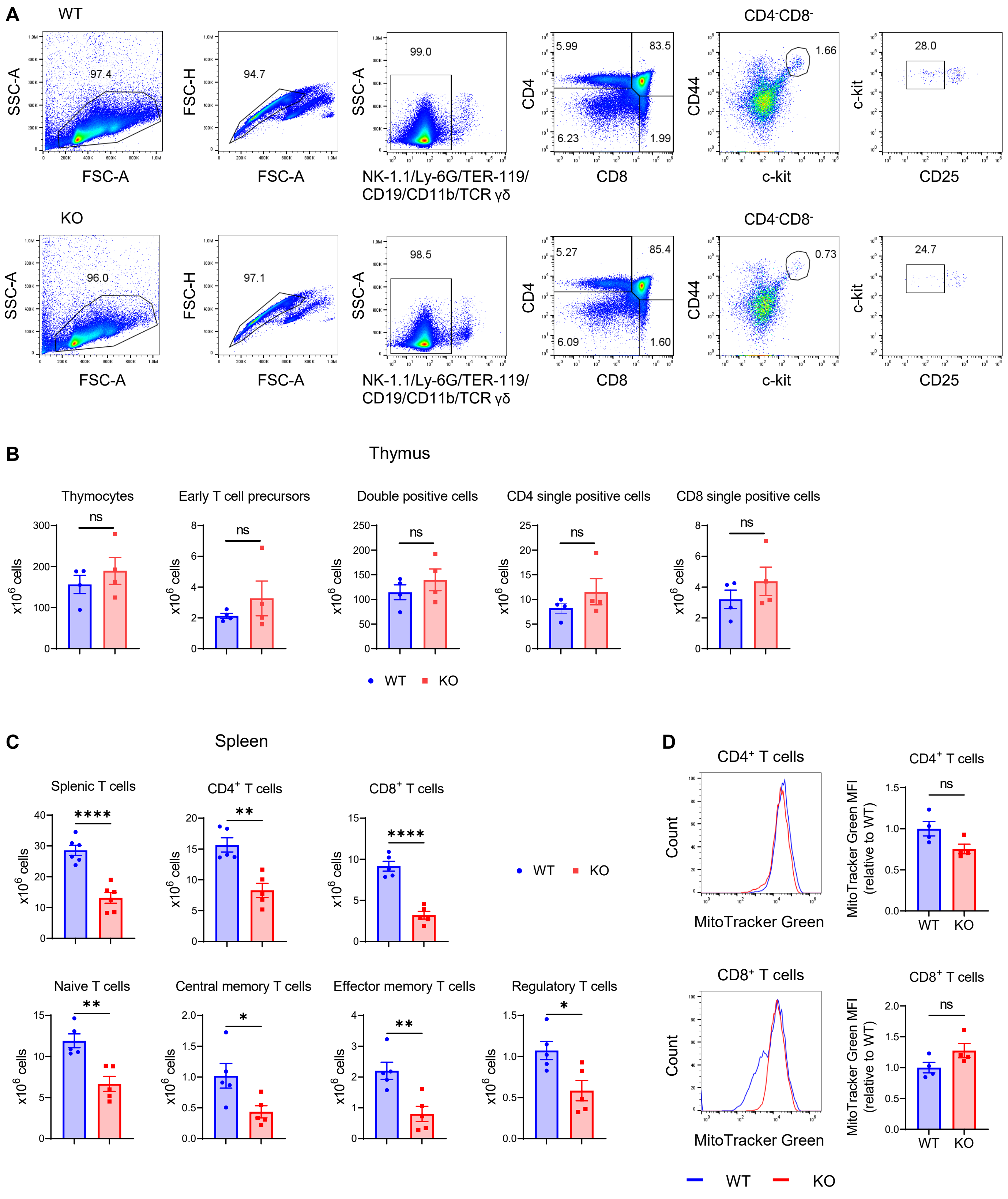

Supplemental Figure 2. SDHA KO showed reduced number of peripheral T cell subsets.

(**A**) Thymocytes were isolated from WT and SDHA KO mice and identified as NK-1.1$^-$Ly-6G$^-$TER-119$^-$CD19$^-$CD11b$^-$TCR γδ$^-$ cells. Within this compartment, CD4$^-$CD8$^-$, CD4$^+$CD8$^+$, CD4$^+$CD8$^-$, and CD4$^-$CD8$^+$ cells were identified as double negative, double positive, CD4 single positive, and CD8 single positive cells, respectively. After identifying double negative cells, early T cell precursors were subsequently identified as CD25$^-$CD44^{high}c-kit^{high}. (**B**) The absolute number of thymus cell subsets in naïve WT and naïve SDHA KO thymus were shown (*n* = 4/group). (**C**) The absolute number of peripheral T cell subsets in naïve WT and naïve SDHA KO splenic T cell were shown (*n* = 5-6/group). (**D**) MitoTracker Green expression levels in WT and SDHA KO T cells stimulated with anti-CD3/CD28 antibodies for 48 h (n = 4/group). 2-tailed unpaired *t* test (**B**-**D**) was used to determine significance (mean ± SEM). *$P < 0.05$, **$P < 0.01$, ****$P < 0.0001$.

Supplemental Figure 3

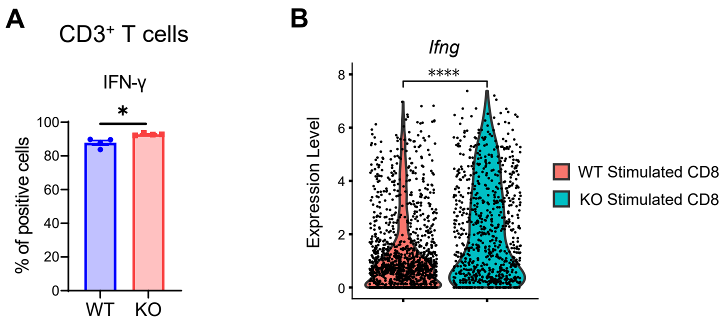

Supplemental Figure 3. SDHA deficiency increased IFN-γ production in T cells after stimulation.

(**A**) Splenic WT and SDHA KO T cells were stimulated by anti CD3/CD28 antibodies for 48 h. IFN-γ positive $CD3^+$ T cells (n = 4/group) were analyzed by flow cytometry. 2-tailed unpaired t test was used to determine significance (mean ± SEM). (**B**) Violin plot showing the expression of *Ifng* in $CD8^+$ T cells stimulated by CD3/CD28 antibodies for 48 h. Wilcox test was used to determine significance. $^*P < 0.05$, $^{****}P < 0.0001$.

Supplemental Figure 4

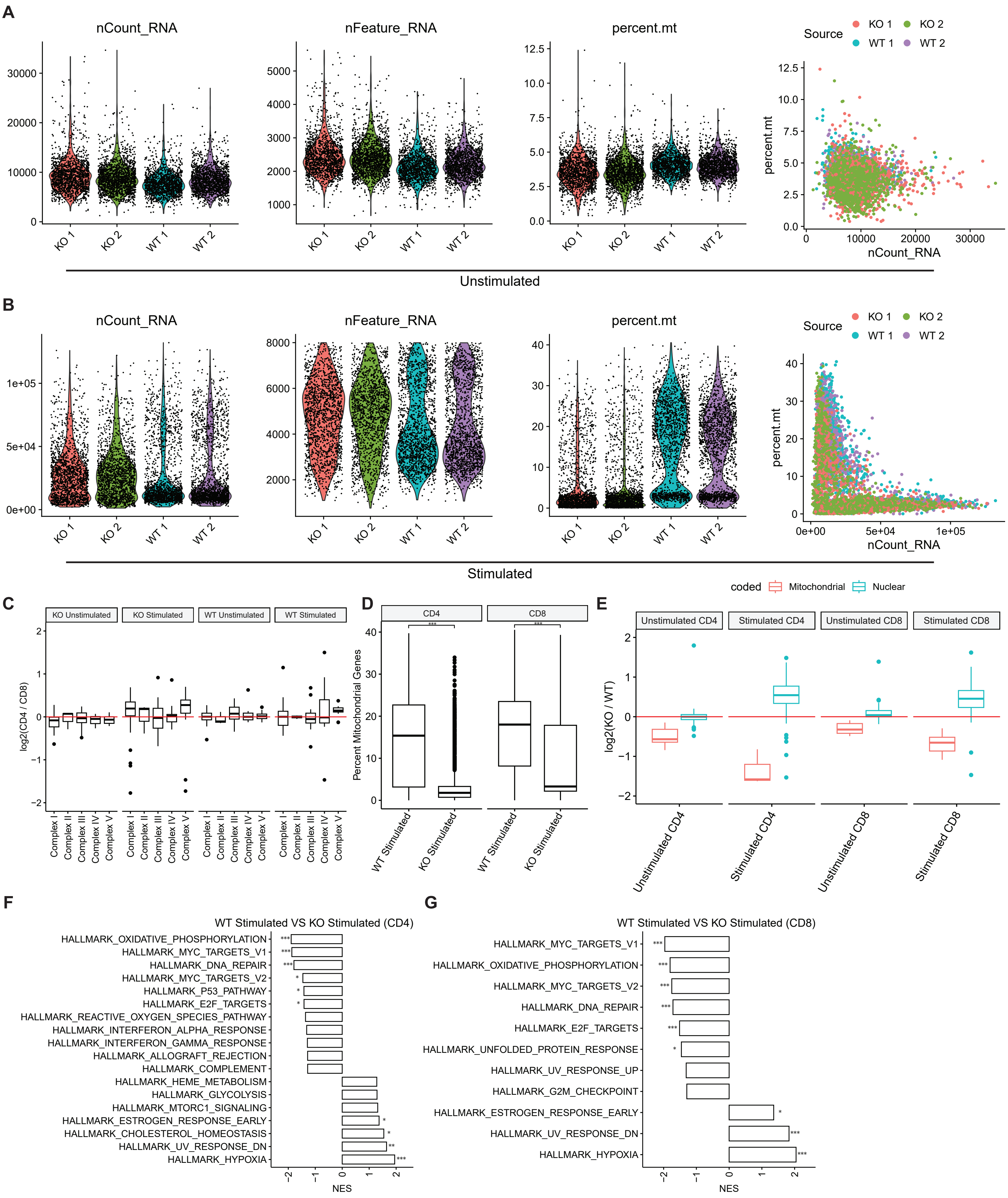

Supplemental Figure 4. scRNA-seq analysis of WT and SDHA KO T cells in unstimulated and stimulated condition.

(**A**) Quality control for unstimulated scRNA-seq samples. (**B**) Quality control for stimulated scRNA-seq samples. Quality controls include number of RNA counts per cell, number of features represented, percent mitochondria reads in cells, and comparison between number of RNA counts and percent mitochondrial reads. (**C**) Fold change of mitochondrial complex genes between $CD4^+$ and $CD8^+$ T cells separated by WT, SDHA KO, stimulated, and unstimulated conditions. (**D**) Percent mitochondrial genes expressed in $CD4^+$ and $CD8^+$ T cells, separated by WT and SDHA KO conditions. (**E**) logFC expressions of mitochondrial complex genes for SDHA KO vs WT across different conditions, separated by mitochondrial-coded or nuclear-coded genes. (**F** and **G**) Hallmark pathway analysis between WT simulated and SDHA KO stimulated samples in $CD4^+$ (**F**) and $CD8^+$ T cells (**G**). Wilcoxon test (**D**) and Permutation test (**F** and **G**) were used to determine significance. $^*P < 0.05$, $^{**}P < 0.01$, $^{***}P < 0.001$.

Supplemental Figure 5

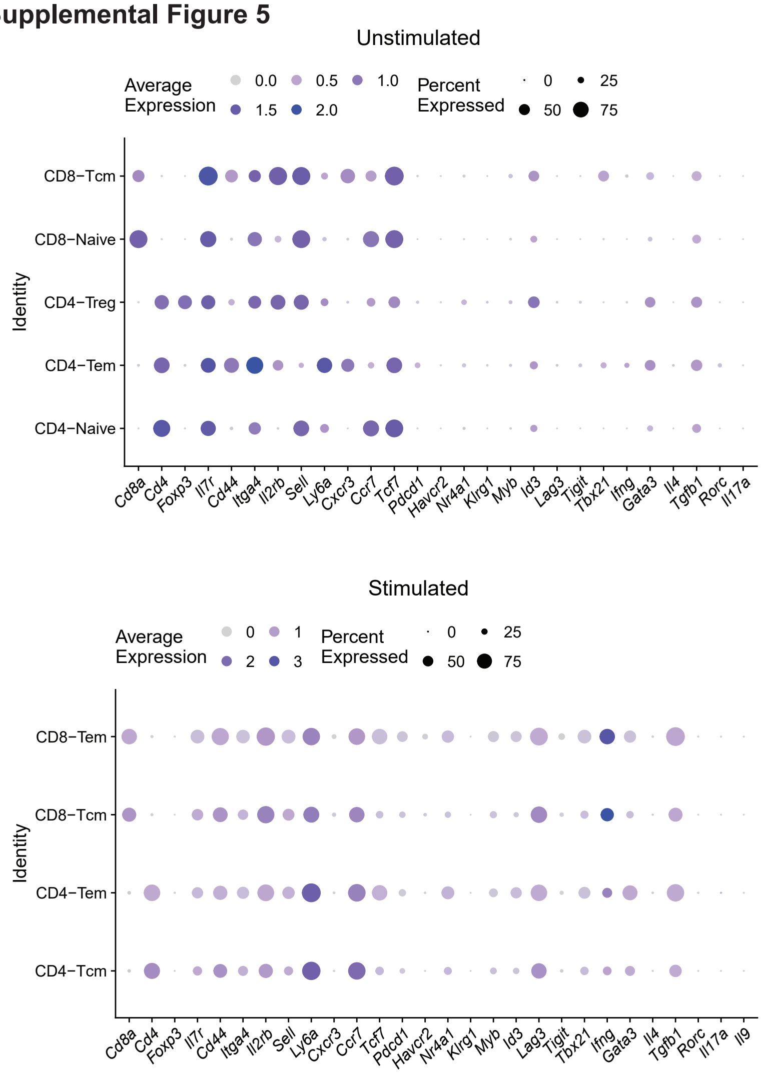

Supplemental Figure 5. Marker expression of T cell clusters in WT and SDHA KO T cells.

Expression of markers related to T-cell differentiation in unstimulated and stimulated T cell subsets.

Supplemental Figure 6

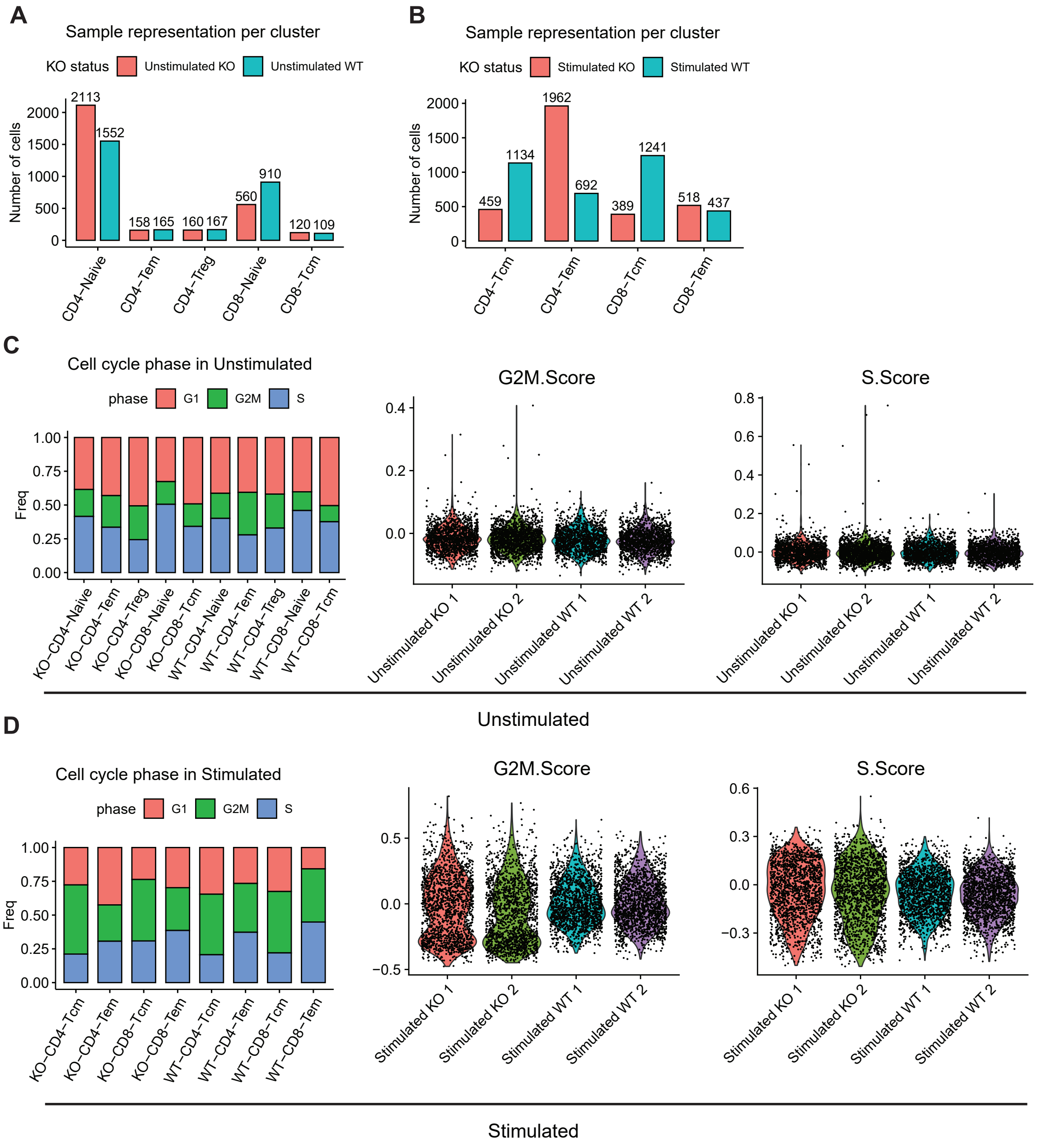

Supplemental Figure 6. SDHA deficiency affects memory T cell differentiation after stimulation.

(**A**) Number and category of T cell subsets in unstimulated WT and SDHA KO T cells. (**B**) Number and category of T cell subsets in stimulated WT and SDHA KO T cells. (**C**) G2M, S phase score and cell cycle phase ratio break down in unstimulated WT and SDHA KO T cells. (**D**) G2M, S phase score and cell cycle phase ratio break down in stimulated WT and SDHA KO T cells.

Supplemental Figure 7

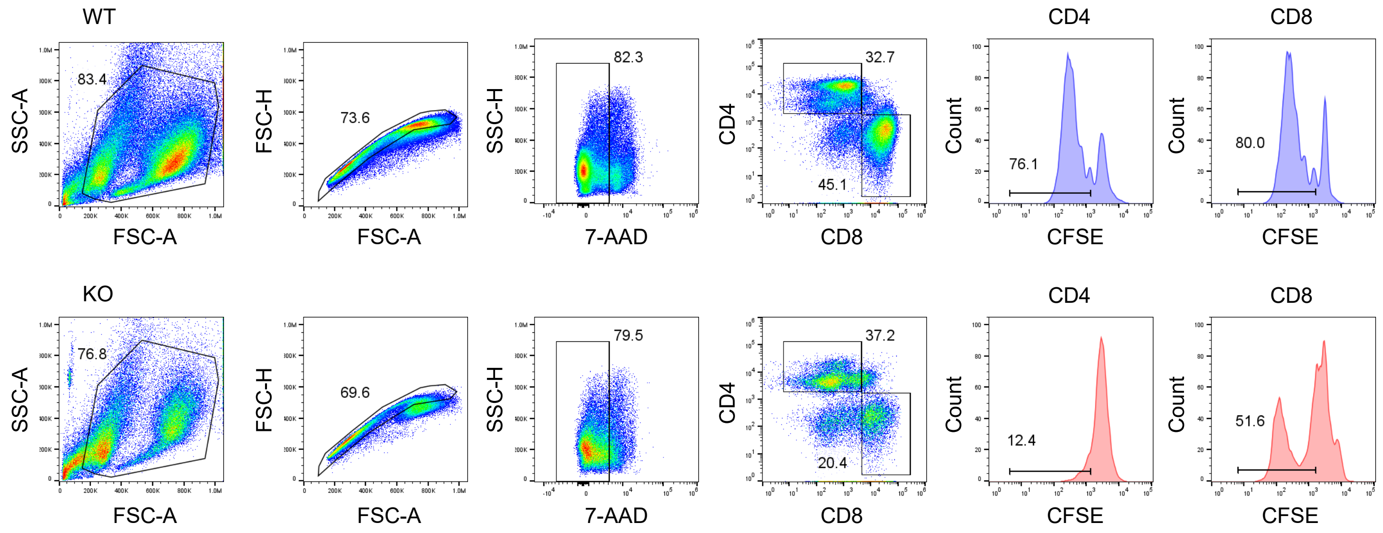

Supplemental Figure 7. Strategy analysis of CFSE dilutions by flow cytometry.

Representative flow cytometry image measuring CFSE dilutions in WT and SDHA KO CD4$^+$ and CD8$^+$ T cells after stimulation with CD3/CD28 antibodies for 48 h.

Supplemental Figure 8

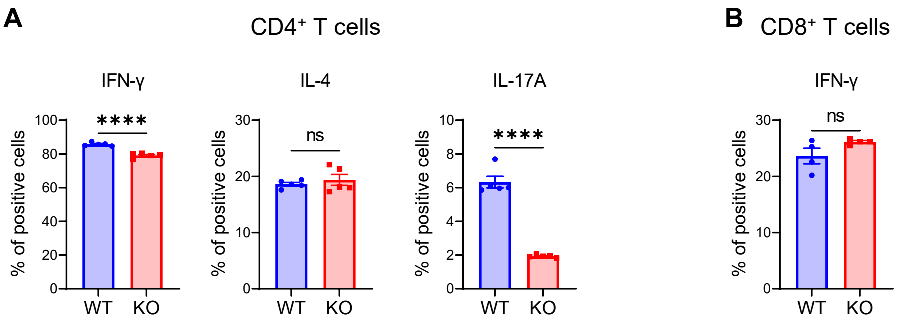

Supplemental Figure 8. SDHA differentially regulates cytokine production in CD4$^+$ and CD8$^+$ T cells.

(**A**) Splenic WT and SDHA KO CD4$^+$ T cells were cultured under conditions of in vitro Th1, Th2, and Th17 polarization. IFN-γ, IL-4, and IL-17A positive CD4$^+$ T cells were analyzed by flow cytometry (*n* = 5/group). (**B**) Splenic WT and SDHA KO CD8$^+$ T cells were stimulated by CD3/CD28 antibodies for 48 h. IFN-γ positive cells were analyzed by flow cytometry (*n* = 4/group). 2-tailed unpaired *t* test (**A** and **B**) was used to determine significance (mean ± SEM). ****P < 0.0001.

Supplemental Figure 9

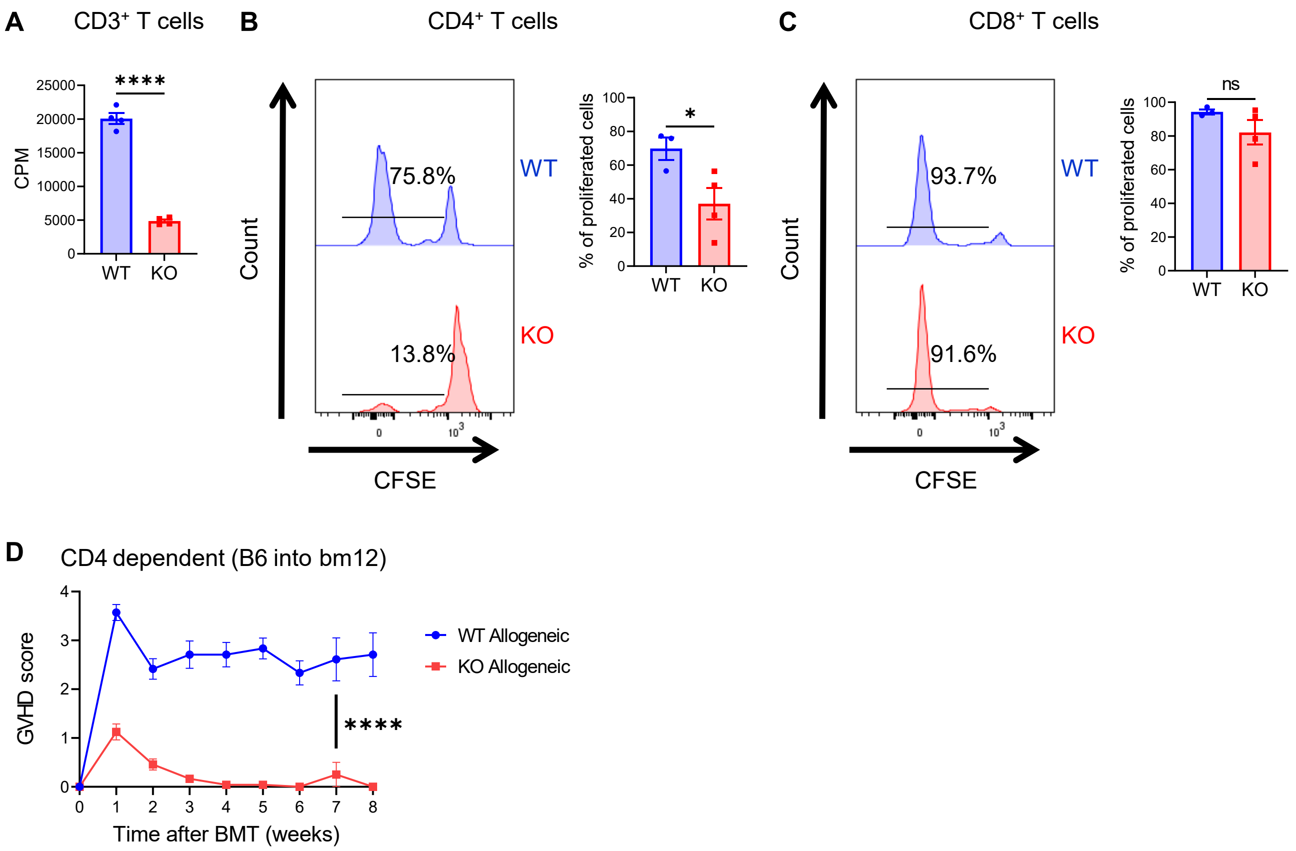

Supplemental Figure 9. The loss of SDHA leads to less proliferation capacity of CD4+ and CD8+ T cells against allogeneic stimulation.

(**A**) WT and SDHA KO T cells were cultured with irradiated BALB/c spleen cells for 72 h. Proliferative capacity was measured by [^{3}H]-thymidine incorporation (n = 4/group). (**B** and **C**) CFSE stained WT and SDHA KO T cells with T cell-depleted bone marrow (TCD-BM) cells were transplanted into BALB/c mice. Proliferative capacity of WT and SDHA KO CD4$^+$ (**B**) and CD8$^+$ (**C**) T cells day 7 after hematopoietic cell transplantation (HCT) were measured by flow cytometry (WT n = 3, KO n = 4). (**D**) bm12 mice received HCT. WT TCD-BM cells with WT T cells or SDHA KO T cells were transplanted. GVHD score was shown (WT n = 15, KO n = 13). 2-tailed unpaired t test (**A-C**) and Mann-Whitney U test (**D**) were used to determine significance (mean ± SEM). *P < 0.05, ****P < 0.0001.

Supplemental Figure 10

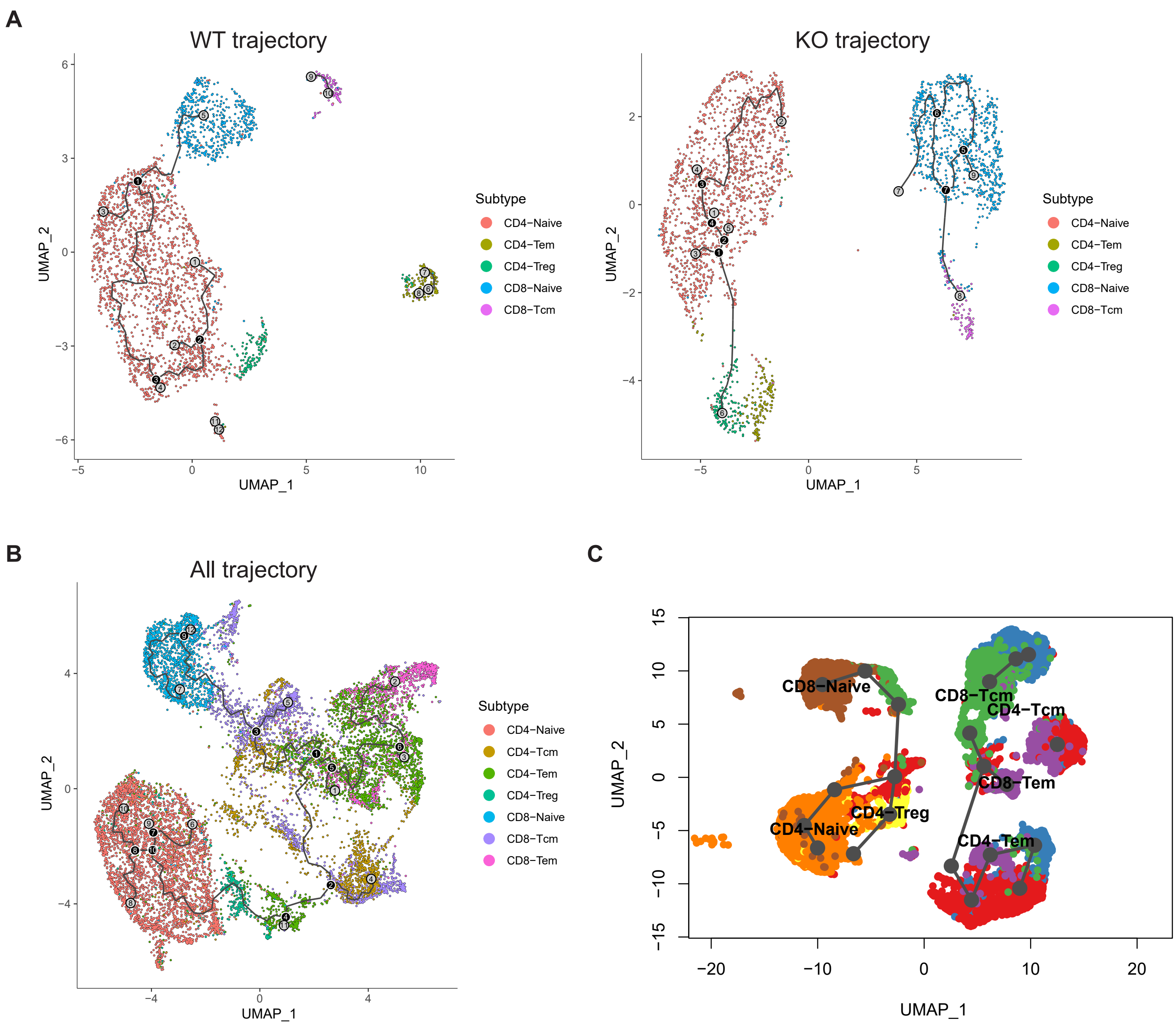

Supplemental Figure 10. Trajectory analysis.

(**A** and **B**) Monocle3 trajectory analysis results using WT and SDHA KO cells separately (**A**), or all cells combined (**B**) are projected onto a UMAP with trajectories marked by connecting nodes and edges. (**C**) Slingshot trajectory analysis utilizing all cells is projected onto a UMAP with trajectories marked by nodes and edges.

Supplemental Figure 11

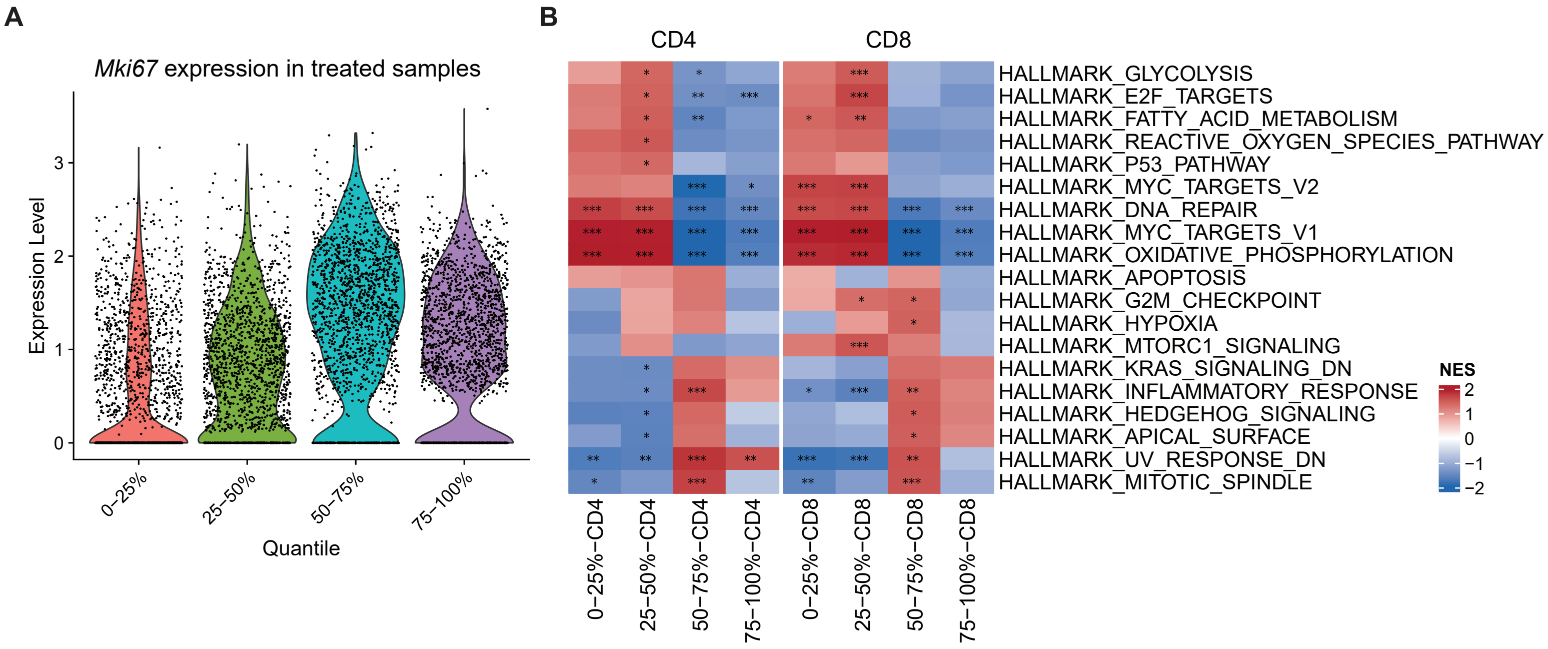

Supplemental Figure 11. Analysis of cells with high mitochondrial content.

(**A**) Violin plot showing expression of proliferation marker *Mki67* across different quantiles of cells. Quantiles are scored based on each cell's percent mitochondrial expression, with 75-100% being cells with the highest percent of mitochondrial expression and 0-25% being the lowest. (**B**) Hallmark pathway analysis of using logFC expression of genes from each quantile against other quantiles, separated by CD4 and CD8 status. Permutation test (**B**) was used to determine significance. **P* < 0.05, ***P* < 0.01, ****P* < 0.001.

Supplemental Table 1. The protocol of HCT model

Day 0: The day of transplantation

HCT model	Recipient	Donor	Conditioning	T cells, cells	TCD-BM, cells
**Figure 5A**	Syngeneic: B6, Allogeneic: BALB/c	WT: *Sdha*$^{fl/fl}$ or KO: *Sdha*$^{fl/fl}$ CD4-Cre	10 Gy for B6 and 8 Gy for BALB/c Total-body irradiation (TBI), day -1	CD90.2$^+$, 2 × 10^6	5 × 10^6
**Figure 5, B-D**	BALB/c	Syngeneic: BALB/c WT Allogeneic: *Sdha*$^{fl/fl}$ KO Allogeneic: *Sdha*$^{fl/fl}$ CD4-Cre	8 Gy TBI, day -1	CD90.2$^+$, 1 × 10^6	5 × 10^6 BM with 1 × 10^3 P815
**Figure 5E**	BALB/c	WT: *Sdha*$^{fl/fl}$ or KO: *Sdha*$^{fl/fl}$ CD4-Cre	8 Gy TBI, day -1	CD90.2$^+$, 2 × 10^6	5 × 10^6
**Figure 5F**	Syngeneic: B6 CD45.1, Allogeneic: BALB/c	WT: *Sdha*$^{fl/fl}$ or KO: *Sdha*$^{fl/fl}$ CD4-Cre	10 Gy for B6 CD45.1 and 8 Gy for BALB/c TBI, day -1	CellTrace Far Red-stained CD90.2$^+$, 2 × 10^6	5 × 10^6
**Figure 5, G and H**	Syngeneic: B6 CD45.1, Allogeneic: BALB/c	WT: *Sdha*$^{fl/fl}$ or KO: *Sdha*$^{fl/fl}$ CD4-Cre	10 Gy for B6 CD45.1 and 8 Gy for BALB/c TBI, day -1	CD90.2$^+$, 2 × 10^6	5 × 10^6
**Supplemental Figure 9, B and C**	BALB/c	WT: *Sdha*$^{fl/fl}$ or KO: *Sdha*$^{fl/fl}$ CD4-Cre	8 Gy TBI, day -1	CFSE-stained CD90.2$^+$, 2 × 10^6	5 × 10^6
**Supplemental Figure 9D**	bm12	WT: *Sdha*$^{fl/fl}$ or KO: *Sdha*$^{fl/fl}$ CD4-Cre	10 Gy TBI, day -1	CD4$^+$, 1.75 × 10^6	5 × 10^6
