# Supplementary material for: Mitochondrial complex II orchestrates divergent effects in CD4+ and CD8+ T cells
Source: J Clin Invest. 2025 Dec 15;135(24):e194134. doi: 10.1172/JCI194134 (PMC12700539; doi:10.1172/JCI194134)

Full unedited gel for Figure 1B

Anti-SDHA antibody (abcam, ab14715)

Splenic T cells

Sdha$^{fl/fl}$ CD4-Cre^{-} *Sdha*$^{fl/fl}$ CD4-Cre^{+}

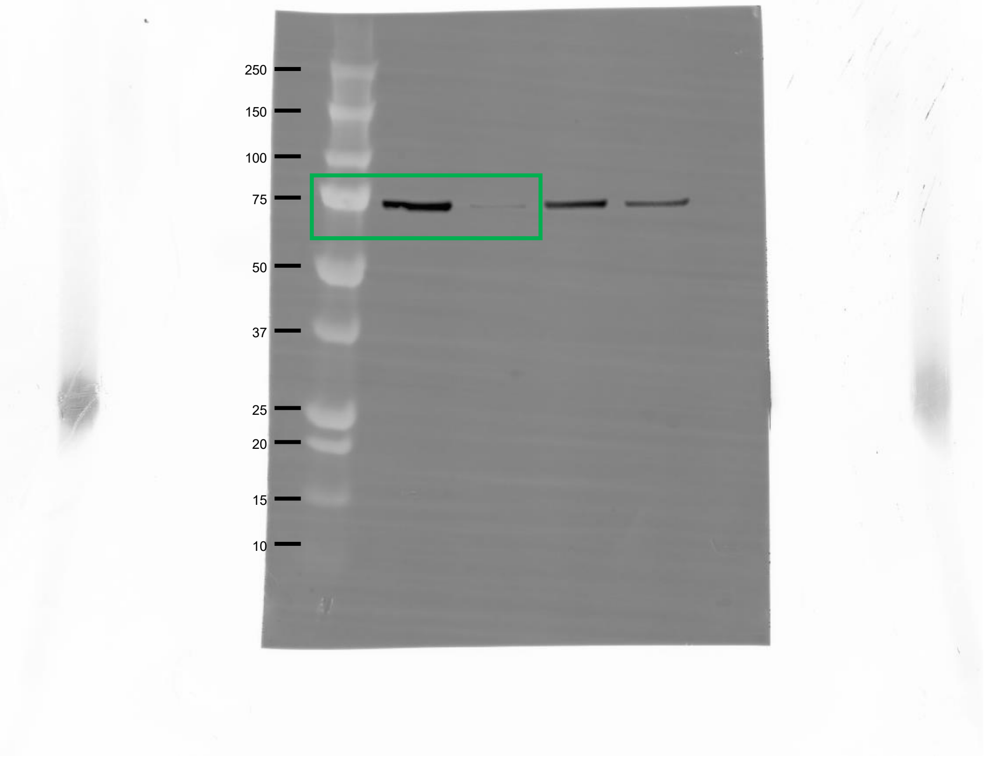

Full unedited gel for Figure 1B

Total OXPHOS Rodent WB Antibody Cocktail (abcam, ab110413)

Splenic T cells

Sdha$^{fl/fl}$ CD4-Cre^{-} *Sdha*$^{fl/fl}$ CD4-Cre^{+}

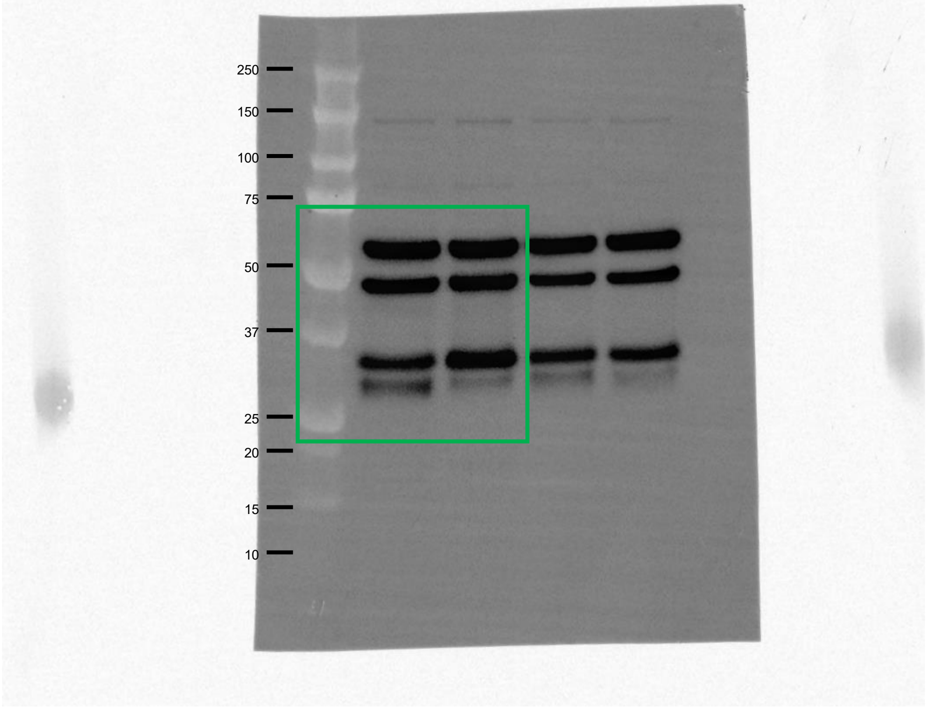

Full unedited gel for Figure 1B

Anti-beta Actin antibody (abcam, ab8226)

Splenic T cells

Sdha$^{fl/fl}$ CD4-Cre^{-} | *Sdha*$^{fl/fl}$ CD4-Cre^{+}

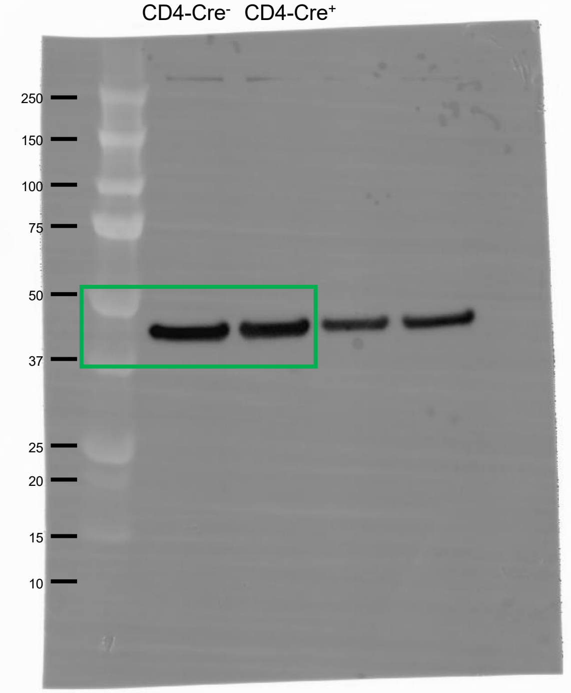

Full unedited gel for Figure 1C

Anti-SDHA antibody (abcam, ab137040)

Sdha$^{fl/fl}$ CD4-Cre^{-} | *Sdha*$^{fl/fl}$ CD4-Cre^{+}

CD4 CD8 CD4 CD8

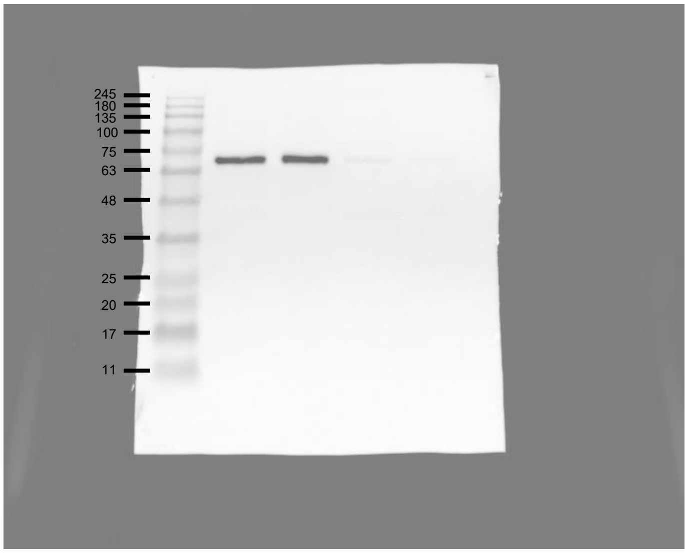

Sdha$^{fl/fl}$ CD4-Cre^{-} | *Sdha*$^{fl/fl}$ CD4-Cre^{+}

CD4 CD4 CD8 CD8 CD4 CD4 CD8 CD8

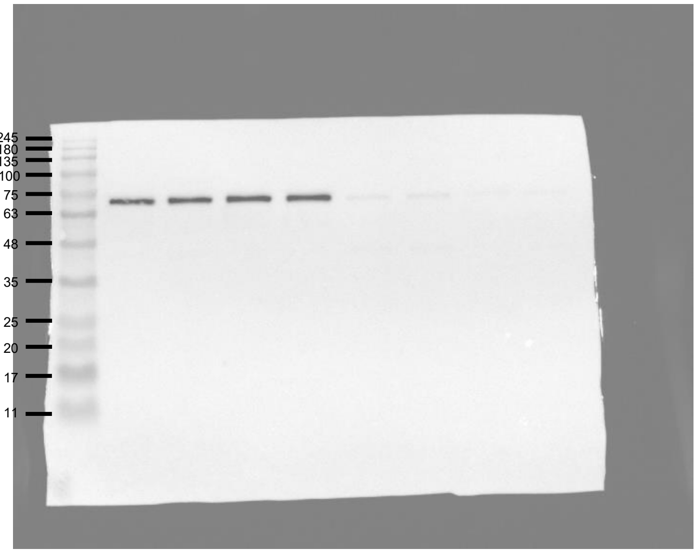

Full unedited gel for Figure 1C

Anti-beta Actin antibody (GeneTex, GTX637675)

Sdha$^{fl/fl}$ CD4-Cre^{-} | *Sdha*$^{fl/fl}$ CD4-Cre^{+}

CD4 CD8 CD4 CD8

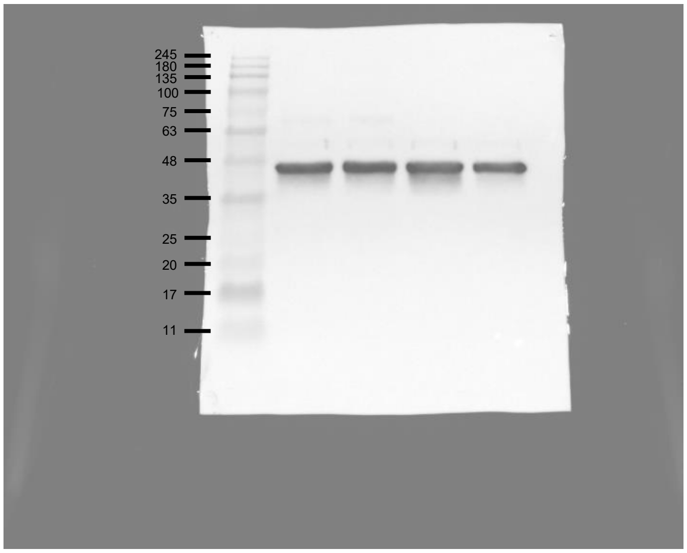

Sdha$^{fl/fl}$ CD4-Cre^{-} | *Sdha*$^{fl/fl}$ CD4-Cre^{+}

CD4 CD4 CD8 CD8 CD4 CD4 CD8 CD8

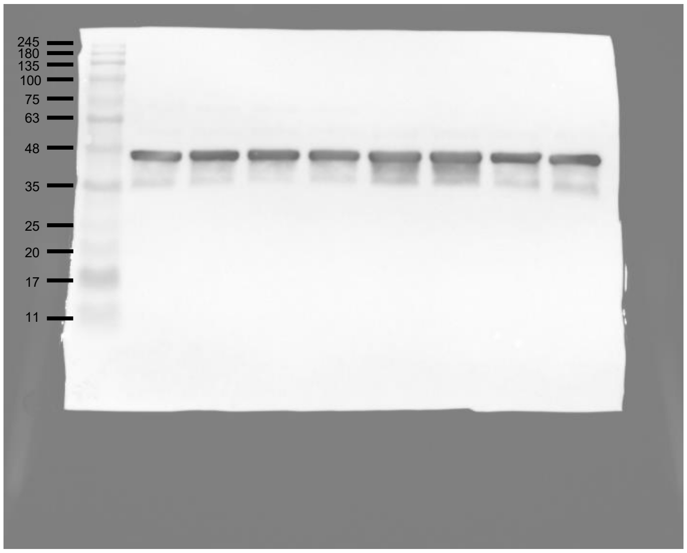

Supplement: Unedited blot and gel images [file jci-135-194134-s084.pdf]
